# A new long-snouted marine reptile from the Middle Triassic of China illuminates pachypleurosauroid evolution

**DOI:** 10.1038/s41598-022-24930-y

**Published:** 2023-01-05

**Authors:** Guang-Hui Xu, Qing-Hua Shang, Wei Wang, Yi Ren, Hong Lei, Jun‑Ling Liao, Li‑Jun Zhao, Chun Li

**Affiliations:** 1 Key Laboratory of Vertebrate Evolution and Human Origins of Chinese Academy of Sciences, Institute of Vertebrate Paleontology and Paleoanthropology, Chinese Academy of Sciences, Beijing, 100044 China; 2 CAS Center for Excellence in Life and Paleoenvironment, Beijing, 100044 China; 3 University of Chinese Academy of Sciences, Beijing, 100049 China; 4 Luoping Biota National Geopark, Land and Resources Bureau of Luoping County, Luoping, 655800 China; 5 College of Economics and Management, Xingyi Normal University for Nationalities, Xingyi, 562400 China; 6 Zhejiang Museum of Natural History, Hangzhou, 310014 China

**Keywords:** Palaeontology, Ecology

## Abstract

Sauropterygia is the largest, most successful group of Mesozoic marine diapsids, spanning from the late Early Triassic to the Late Cretaceous. Plesiomorphic for sauropterygians, pachypleurosauroids are important for our understanding on the early evolution of this group. Here, we present a new pachypleurosaurid, *Luopingosaurus imparilis* gen. et sp. nov., based on an exceptionally preserved skeleton from the early Middle Triassic Luoping Lagerstätte in Yunnan, China. The discovery documents the first long-snouted pachypleurosaurid with an unexpected hyperphalangy in the manus, providing new insights into the morphological diversification, ecological adaption and biogeographic evolution of this clade. The discovery further indicates that there is a morphological divergence between short-snouted (brevirostrine) keichousaurids and relatively long-snouted (longirostrine) pachypleurosaurids, which was probably driven by ecological specializations related to feeding and foraging. Additionally, an evolutionary trend towards the reduction of the ratio of the hyoid length to mandibular length (HM ratio) is recognized in pachypleurosauroids. This reduction of HM ratio, associated with the increase of the snout length, might implicate a gradual recession of suction feeding in pachypleurosauroid evolution. Phylogenetic studies incorporating *Luopingosaurus* recover European pachypleurosaurids as successive sister groups to Chinese derived pachypleurosaurids, supporting a western Tethyan origin for this family.

## Introduction

Sauropterygia is the largest, most taxonomically rich group of Mesozoic marine diapsids that is composed of two major subgroups, Placodontia and Eosauropterygia^1,2^. The lizard-like pachypleurosauroids are small to medium-sized eosauropterygians inhabited the epicontinental seas and intraplatform basins of the Tethyan Ocean in the Early to Middle Triassic^3,4^. The pachypleurosauroid evolution represents an important case of early adaptive radiation of sauropterygians in the aftermath of the end-Permian mass extinction^4,5^. Pachypleurosauroid fossils have long attracted the attention of palaeontologists interested in early sauropterygian evolution because they remain plesiomorphic for this group and provide a basis for our understanding on the transition between primitive terrestrial diapsids and fully aquatic eusauropterygians (including nothosauroids, pistosaurids and plesiosaurids)^6–16^.

Our understanding of the pachypleurosauroid evolution continues to improve in recent years, driven predominantly by new findings from South China^17–23^, Europe^24,25^ and Myanmar^26^. The earliest known pachypleurosauroids can now be traced to the late Early Triassic in South China^17^, and in the Middle Triassic, pachypleurosauroids underwent a rapid radiation, represented so far by seven genera from Europe^25^, nine genera from South China^23^, and unnamed taxa from Myanmar^26^. The relationships of pachypleurosauroids with other sauropterygian clades remain debated. Pachypleurosauroids are recently recovered as the sister group of Eusauropterygia (Nothosauroidea and Pistosauroidea) or of Nothosauroidea^14,21–23,27,28^; additionally, they fall into an unresolved grade within Eosauropterygia in some analyses^29^. It was traditionally considered that pachypleurosaurids were geologically confined to the Middle Triassic of Europe and Chinese pachypleurosauroids (keichousaurids and other pachypleurosaurid-like forms) were more plesiomorphic than their Europe relatives^14,27,28^. However, recent analyses^21–23^ indicate that some Chinese pachypleurosauroids (e.g., *Qianxisaurus*^16^ and *Honghesaurus*^23^) are likely direct descendants of European pachypleurosaurids, and the biogeographic evolution of pachypleurosauroids was probably more complicated than the previously thought^27^.

**Figure 1:**
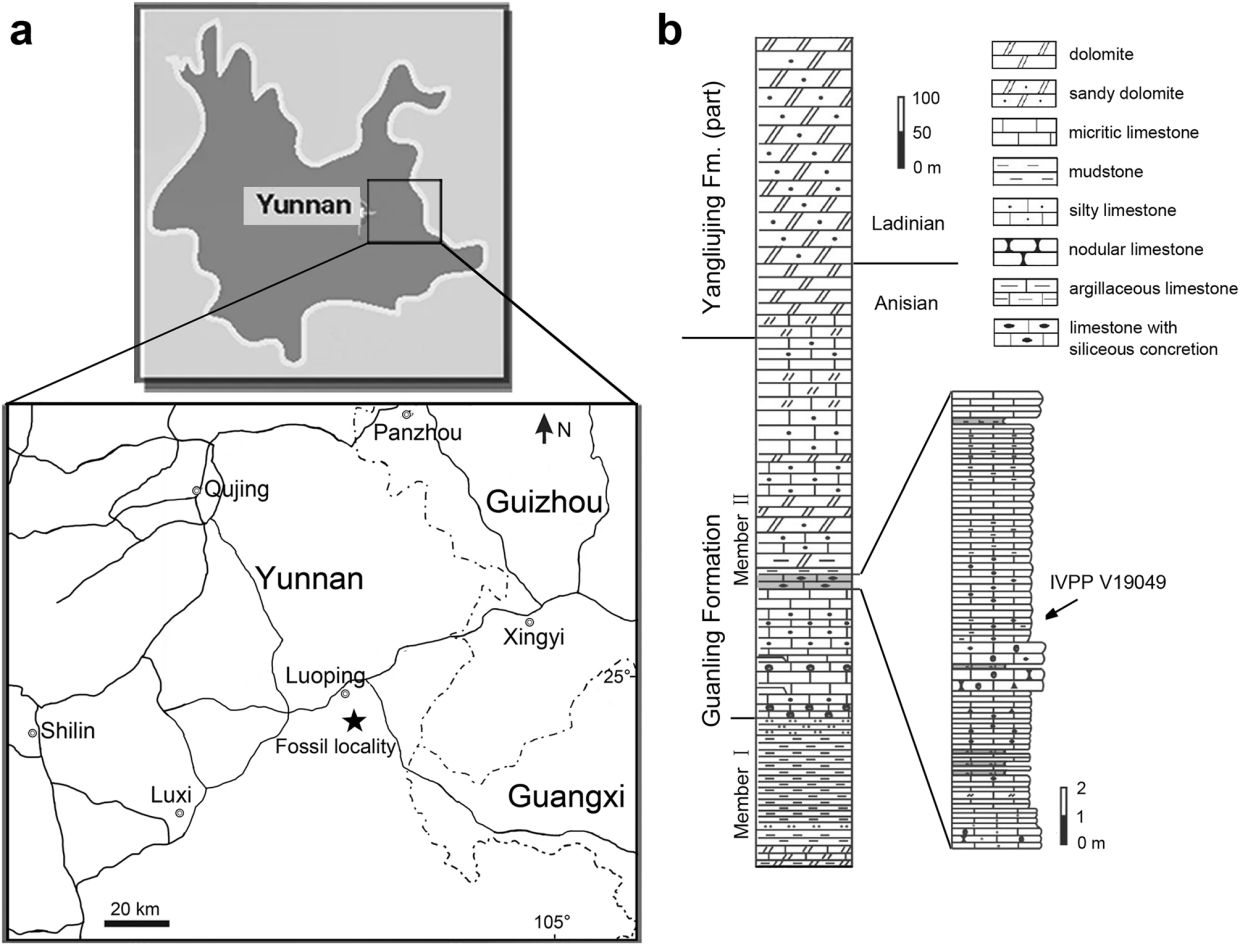
Locality map and stratigraphic section. The fossil locality in Luoping, eastern Yunnan (**a**) is indicated by a star, and the stratigraphic section in Luoping (**b**) is modified from ref^33^.

**Figure 2:**
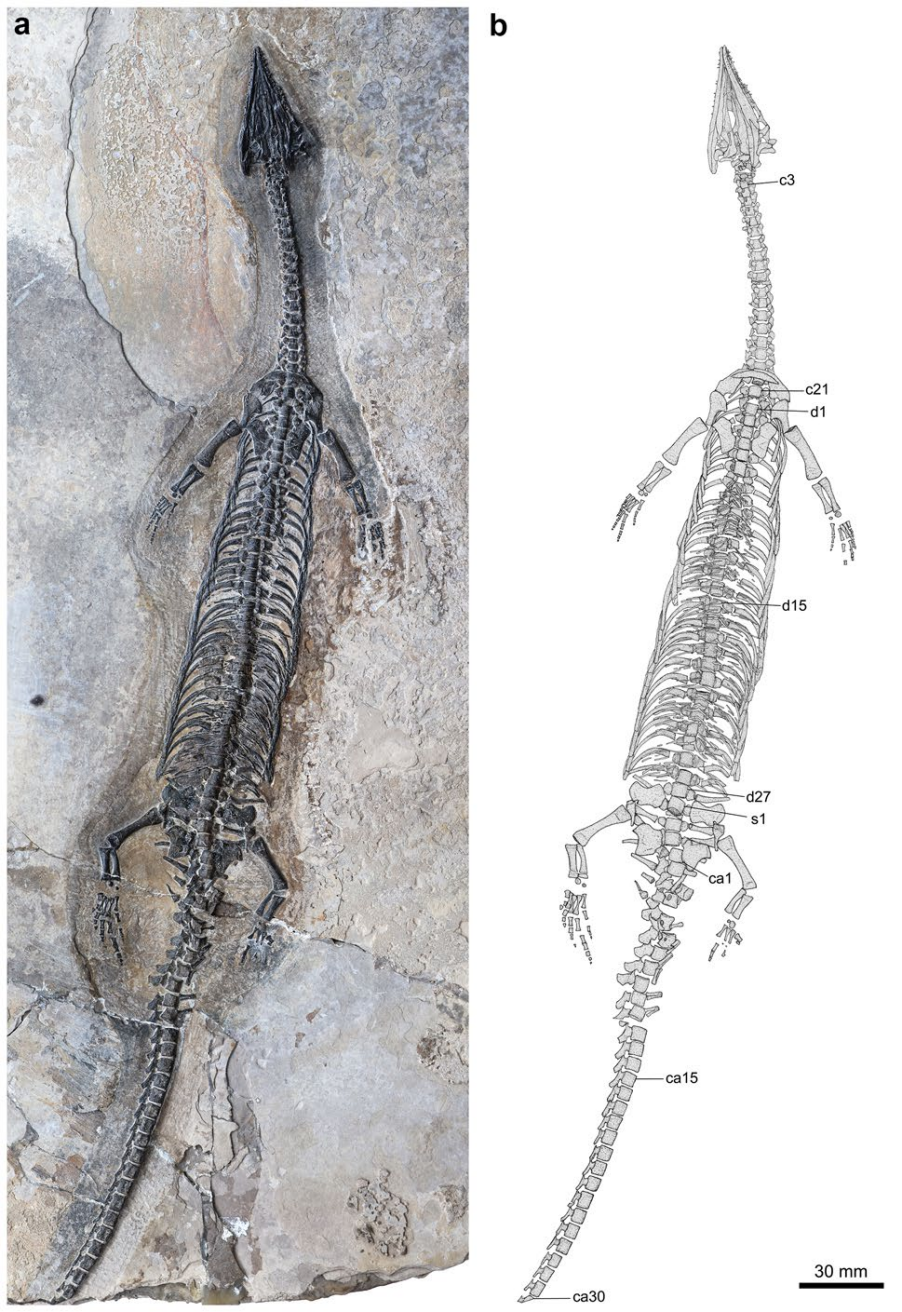
*Luopingosaurus imparilis* gen. et sp. nov., Holotype (IVPP V19049). Photo (**a**) and line-drawing (**b**) of whole specimen. c, cervical vertebra; ca, caudal vertebra; d, dorsal vertebra; s, sacral vertebra.

Here, we report the discovery of a sauropterygian marine reptile on the basis of an exceptionally well-preserved skeleton from the Second (Upper) Member of the Guanling Formation exposed in Luoping, Yunnan, China (Fig. 1). The skeleton is ventrally exposed in a large slab (367 mm × 670 mm) of micritic limestone with a posterior part of the tail missing (Fig. 2). The marine reptile displays a suite of morphological characters identifying it as a new pachypleurosaurid from the Middle Triassic Luoping Biota (or Lagerstätte). With a preorbital length measuring 55% of the skull length, the new taxon represents the first long-snouted pachypleurosaurid from Yunnan, which provides new insights into the morphological diversity and ecological adaption of this clade. The only previously known pachypleurosaurid with an even longer snout is the slightly younger *Wumengosaurus* from Panzhou in Guizhou Province^13,30^, and this enigmatic pachypleurosaurid was once regarded as a close relative of Ichthyosauromorpha^31,32^. The discovery of a similarly long-snouted pachypleurosaurid from Luoping stimulates a comprehensive analysis based on an updated dataset to resolve the phylogenetic relationships of these pachypleurosauroids with other major clades of marine reptiles, and to discuss the evolution of key characters in pachypleurosauroids. Besides the new pachypleurosaurid, other animals known from the fossiliferous beds in the Luoping localities include taxonomically rich invertebrates, fishes, two mixosaurids, two saurosphargids, an incertae sedis sauropterygian, two nothosaurids and several other pachypleurosauroids^5,14,15,18,22,33–36^. The age (Pelsonian, Anisian, ~ 244 Ma) of the fossil beds is well constrained by conodont biostratigraphy^37^. Consequently, the Luoping Lagerstätte provides a unique window into the recovery and radiation of marine ecosystems ~ 8 Myr after the end-Permian mass extinction^5^.

## Results

### Systematic paleontology

Sauropterygia Owen, 1860^38^.

Eosauropterygia Rieppel, 1994^39^.

Pachypleurosauroidea Huene, 1956^40^.

Pachypleurosauridae Nopcsa, 1928^41^.

*Luopingosaurus imparilis* gen. et sp. nov.

### Etymology

The genus name refers to the Luoping County, at which the fossil site is located. Species epithet *imparilis* (Latin) means peculiar and unusual.

### Holotype

A ventrally exposed skeleton with a posterior part of the caudal missing, IVPP V19049.

### Locality and horizon

Luoping, Yunnan, China; Second (Upper) Member of Guanling Formation, Pelsonian (~ 244 Ma), Anisian, Middle Triassic^37^.

### Diagnosis

A pachypleurosaurid distinguishable from other members of this family by the following combination of features (those unique among pachypleurosaurids identified with an asterisk): snout (preorbital portion) long and anteriorly pointed, 55.0% of skull length (*); orbital length about one quarter of skull length; internal naris retracted, without contribution from premaxilla; nasal ending at level of anterior margin of prefrontal; dentary length 71.7% of mandibular length; hyoid length 9.7% of mandibular length; presence of entepicondylar foramen in humerus; 21 cervical and 27 dorsal vertebrae (*); distinct expansions of distal heads of posterior two sacral ribs; six pairs of caudal ribs; phalangeal formula 2–3-5–5-3 for manus and 2–3-4–6-4 for pes (*); Metatarsal I short and stout with expanded proximal end, 56.4% of Metatarsal V in length (*); and Metatarsal IV being longest phalange in pes.

### Comparative description

The holotype and only currently known specimen of *Luopingosaurus* has a preserved length of 46.2 cm from the rostral tip to the 30th caudal vertebra (for measurements, see Table 1). The estimated total length of the body may have reached 64 cm, assuming similar tail proportions of pachypleurosaurids. As such, *Luopingosaurus* is longer than most of other pachypleurosauroids that are small-sized with a maximum total length rarely exceeding 50 cm^4,9–12,14–16,18,23,25^, although some pachypleurosaurids are notably larger (e.g., 88 cm in *Diandongosaurus* cf. *acutidentatus*^22^, ~ 120 cm in *Neusticosaurus edwardsii*^8^, and ~ 130 cm in *Wumengosaurus delicatomandibularis*^13^).

**Table 1:** Measurements (in mm) of the holotype (IVPP V19049) of *Luopingosaurus imparilis* gen. et sp. nov. R, right.

Total length	461.9+
Snout length	23.5
Skull length (premaxillary symphysis to occipital condyle)	42.7
Length of orbit	10.6
Length of axis to last cervical vertebra	80.2
Length of axis to last dorsal vertebra	225.2
Length of mandible	46.2 (R)
Length of humerus	19.8 (R)
Proximal width of humerus	5.5 (R)
Distal width of humerus	6.0 (R)
Length of radius	12.2 (R)
Length of ulna	10.9 (R)
Length of metacarpal I	2.2 (R)
II	4.6 (R)
III	5.3 (R)
IV	5.5 (R)
V	3.9 (R)
Length of femur	20.8 (R)
Proximal width of femur	6.1 (R)
Distal width of femur	5.9 (R)
Length of fibula	12.3 (R)
Length of tibia	11.6 (R)
Length of metatarsal I	3.1 (R)
II	6.1 (R)
III	6.9 (R)
IV	7.2 (R)
V	5.5 (R)

**Figure 3:**
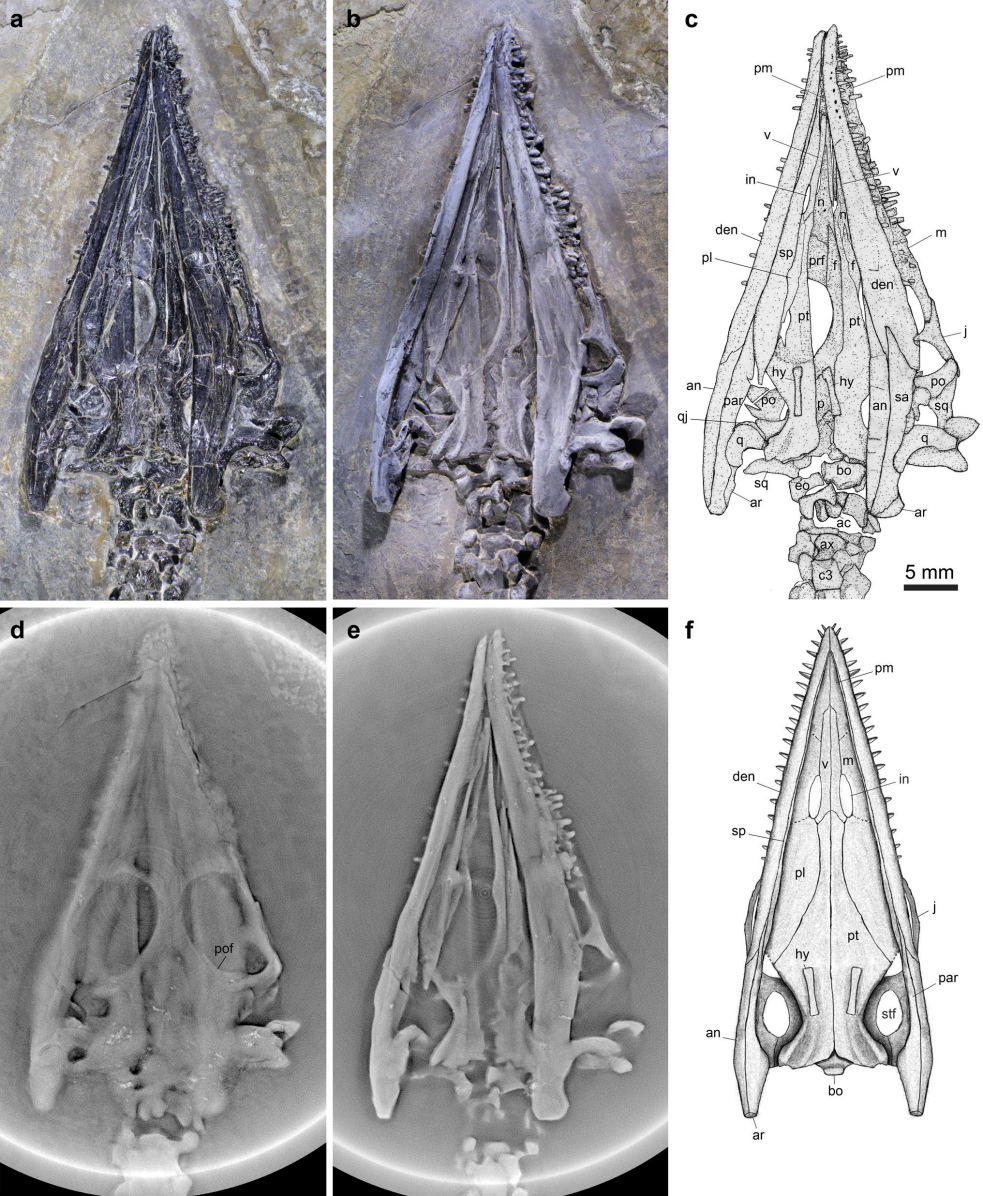
Skull and mandible of *Luopingosaurus imparilis* gen. et sp. nov., IVPP V19049. Head before (**a**) and after (**b**) dusted with ammonium chloride. (**c**), Line- drawing. (**d**, **e**), two computed laminography scanning slices. (**f**), reconstruction in ventral view. ac, acetabulum; an, angular; ar, articular; ax, axis; c, cervical vertebra; den, dentary; eo, exoccipital; f, frontal; hy, hyoid; in, internal naris; j, jugal; m, maxilla; n, nasal; p, parietal; par, prearticular; pof, postfrontal; prf, prefrontal; pt, pterygoid; q, quadrate; qj, quadratojugal; sa, surangular; sp, splenial; sq, squamosal; stf, supratemporal fossa; v, vomer.

The pre-orbital portion, distinctly longer than the postorbital region, measures 55% of the total skull length (the premaxillary symphysis to the occipital condyle) and 51% of the mandibular length. The paired premaxillae form most of the snout anterior to the naris with a pointed anterior tip, similar to the conditions in *Wumengosaurus*^13,30^ and *Honghesaurus*^23^. By contrast, other pachypleurosauroids uniformly have a blunt rostrum^4,6–12,14–16,18,22,25^. The premaxilla bears a long posteromedial process inserting between the anterior parts of the elongate nasals (Fig. 3). The premaxillary teeth are homodont with a tall peduncle and a short, conical crown, but the tooth number is hard to estimate because of occlusion of jaws. The posterior parts of nasals contact each other medially, and posteriorly, they contact the frontals in an interdigitating suture at the level of the anterior margin of the prefrontal. In *Honghesaurus*^23^, *Wumengosaurus*^30^, *Neusticosaurus*^8^ and *Serpianosaurus*^9^, the even longer nasal extends posteriorly beyond this level and ends at the anterior portion of the orbit.

The orbit is oval and large, measuring 24.8% of the skull length (Fig. 3). The lateral margin of the frontal contacts the prefrontal anteriorly and the postfrontal posteriorly, and defines most of the medial border of the orbit. The L-shaped jugal, together with the posterolateral process of the maxilla, forms the lateral border of the orbit. No distinct lacrimal is discernable; the bone is probably absent as in other sauropterygians. The postfrontal contacts the dorsal process of the triradiate postorbital ventrally, and both bones define the posterior border of the orbit. Additionally, the posterior process of the postorbital contacts the anterior process of the squamosal, forming the bar between the supratemporal fossa and the ventrally open infratemporal fenestra. The jugal extends beyond the ventral margin of the postorbital and also contacts the anterior process of the squamosal, resembling the conditions in *Wumengosaurus*^30^, *Honghesaurus*^23^ and *Diandongosaurus*^15^. This contact is absent in other pachypleurosauroids^4,6–12^.

A pair of vomers and pterygoids and a right palatine are discernable in the palate (Fig. 3a–c). The vomer is elongate and slender, extending anteriorly well beyond the nasal. The internal naris, partly covered by the detached splenial, is longitudinally retracted, corresponding to a retracted external naris (Fig. 3d–f). The medial margin of the naris is defined by the nasal, without contribution from the premaxilla. A retracted naris is otherwise present in *Wumengosaurus*^13,30^, *Qianxisaurus*^16^ and *Honghesaurus*^23^. Similar to the condition in *Honghesaurus*^23^, the retracted naris of *Luopingosaurus* is relatively short, having a longitudinal diameter distinctly less than half of the longitudinal diameter of the orbit. By contrast, other pachypleurosauroids^4,6–12,25^ generally have an oval-shaped naris. The elongate palatine has a slightly convex medial margin suturing with the pterygoid. Because of the coverage of the detached splenial, the lateral portion of the palatine is unexposed, and it is hard to know whether an ectopterygoid is present or not. The pterygoid is the largest and longest element of the palate, measuring 55.2% of the mandibular length. It has an anterior projection that contacts the vomer anteromedially, and does not participate in the margin of the internal naris. At the level of the posterior orbital margin, the pterygoid has a triangular lateral extension, which was termed as the ectopterygoid process of the pterygoid in *Neusticosaurus*^8^. The pterygoid extends back to the occipital condyle, and covers the basicranium and parietals in ventral view. Additionally, the bone has a broad posterolateral process that is set off from the palatal surface by a distinct ridge, resembling the conditions in *Serpianosaurus*^9^ and *Neusticosaurus*^8^. Posteriorly, the basioccipital is exposed in ventral view, showing the area for attachment to the right exoccipital.

The left quadrate is exposed in lateral view with its dorsal process extending underneath the base of the descending process of the squamosal. The posterior margin of the quadrate is excavated, as in many other pachypleurosaurids (e.g., *Serpianosaurus*^9^ and *Honghesaurus*^23^). The quadratojugal is narrow and splint-like, flanking the anterior margin of the quadrate. A pair of hyoids are ossified. They are rod-like, slightly expanded at both ends. The dentary is wedge-shaped, being 71.7% of the mandibular length. Laterally, it bears a longitudinal series of pores and grooves parallel to the oral margin of the bone (Fig. 3a). The elongate angular tapers at both ends, contacting the dentary anterodorsally and the surangular dorsally in ventral view. The surangular, slightly shorter than the angular, contacts the articular posterodorsally, with a pointed anterior tip wedging into the notched posterior margin of the dentary. The retroarticular process of the articular is very short with a rounded posterior margin. Medially, the splenial and prearticular form most of the inner wall of the mandible. The splenial tapers at both ends and enters the mandibular symphysis anteriorly, having a length similar to the dentary. The relatively slender prearticular contacts the splenial anterodorsally, extends posteriorly and abuts the articular dorsally, measuring 41.1% of the mandibular length.

**Figure 4:**
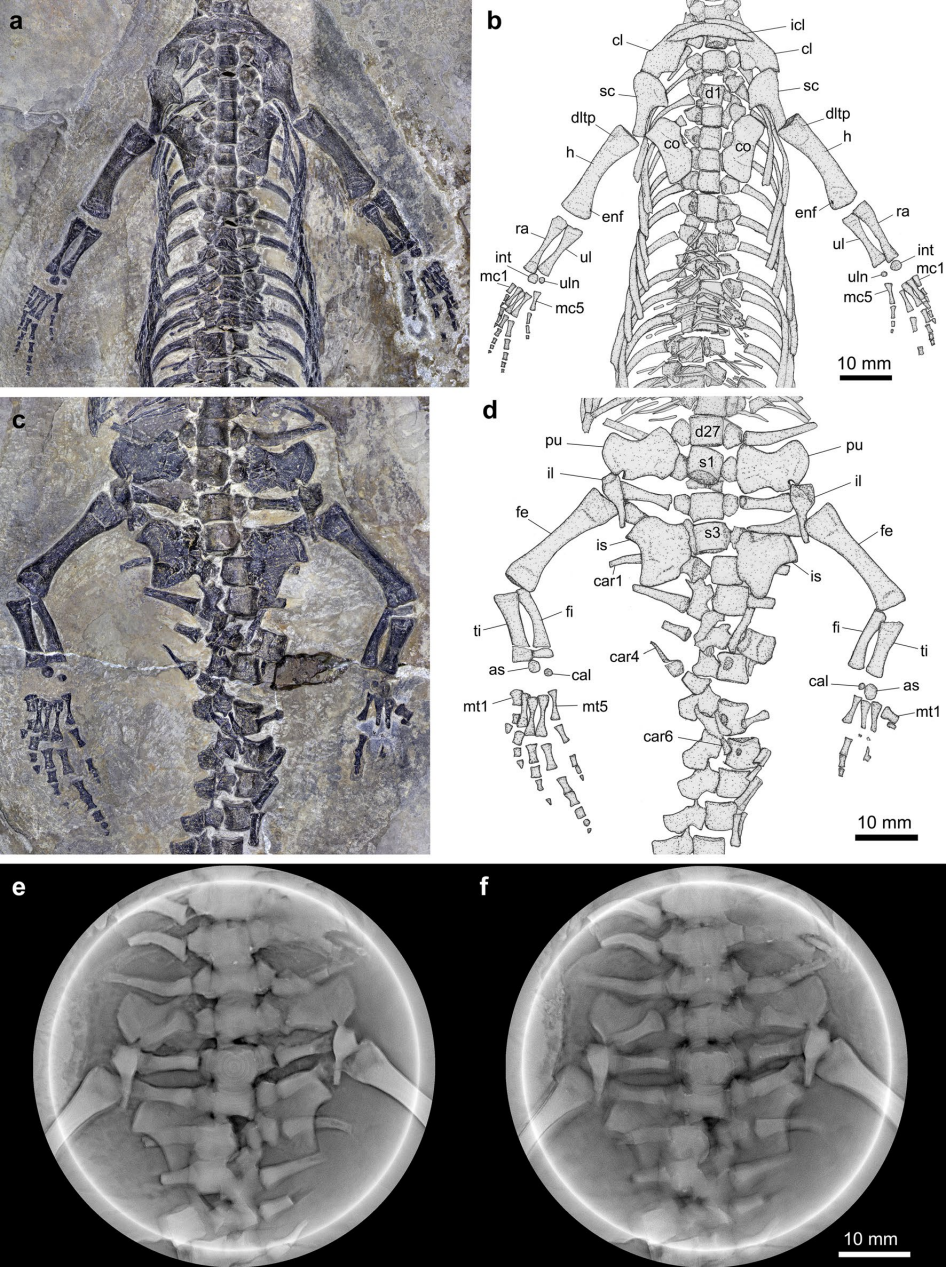
Girdles, limbs and vertebrae of *Luopingosaurus imparilis* gen. et sp. nov., IVPP V19049. Photo (**a**) and line-drawing (**b**) of pectoral girdle, forelimbs and anterior dorsal vertebrae. Photo (**c**), line-drawing (**d**) and two computed laminography scanning slices (**e**, **f**) of pelvic girdle, hind limbs and posterior vertebrae. as, astragalus; ca, caudal vertebra; cal, calcaneum; car, caudal rib; co, coracoid; d, dorsal vertebra; dltp, deltopectoral crest; enf, entepicondylar foramen; fe, femur; fi, fibula; h, humerus; il, ilium; int, intermedium; is, ischium; mc, metacarpal; mt, metatarsal; pu, pubis; s, sacral vertebra; sc, scapula; sr, sacral rib; ti, tibia; ul, ulna; uln, ulnare.

The whole series of 21 cervical vertebrae (including the atlas-axis complex) is well exposed ventrally. The atlas centrum is oval, much smaller than the axis centrum (Fig. 3c). From the axis, the cervical vertebrae increase gradually in size toward the trunk vertebrae posteriorly. The bicephalous cervical ribs have typical free anterior and posterior processes as in other pachypleurosauroids^8,9^. The trunk is relatively long, including 27 dorsal vertebrae. The posterior dorsal ribs show certain pachyostosis (Fig. S1). Each gastralium consists of five elements (a short and more massive median element and two slender rods in line towards each side; Figs. 3, 4a, b, S1), similar to the conditions in most of other pachypleurosauroids^9,11,18,25^ (except *Neusticosaurus*^8^). Three sacral ribs are clearly revealed by X-ray computed microtomography (Fig. 4c–f). They are relatively short and stout, with the posterior twos bearing a distinct expansion on their distal heads. The distal expansion of the sacral rib is also present in *Keichousaurus*^11^, *Prosantosaurus*^25^, *Qianxisaurus*^16^ and *Wumengosaurus*^13^, but it is not pronounced in other pachypleurosauroids^4,6–10^. The caudal ribs are relatively few, six pairs in number. Additionally, several chevron bones are visible in the proximal caudal region, and they are gradually reduced in length posteriorly (Fig. 4d).

The paired clavicles and the median interclavicle form a transverse bar at the 20th cervical vertebrae (Fig. 4a, b). The blade-like clavicle tapers posterolaterally with its distal projection overlapped by the scapula in ventral view. The left clavicle contacts the right one anterodorsally to the interclavicle. The interclavicle tapers laterally to a point at each end. The anterior margin of the interclavicle is convex and its posterior margin is slightly concave without a midline projection (contra the condition in *Anarosaurus*^42^). The scapula consists of a broad ventral portion and a relatively narrow and elongate dorsal wing that varies little through its length. The coracoid is hourglass-shaped with a slightly concave posterolateral margin and a conspicuously concave anteromedial margin. The medial margin is straight, along which the coracoids would articulate each other in the midline. The humerus is constricted at the middle portion with a nearly straight preaxial margin and a concave postaxial margin. A slit in the expanded distal portion of this bone indicates the possible presence of an entepicondylar foramen (Fig. 4a, b). The radius, slightly longer than the ulna, is more expanded proximally than distally. The ulna is straight with a slightly constricted shaft. In each forelimb, there is two nearly rounded carpals, ulnare and intermedium; the former is half the width of the latter. Five metacarpals are rod-like, slightly expanded at both ends. Among them, Metacarpal I is the shortest, 48% of the length of Metacarpal II. Metacarpal III is slightly shorter than Metacarpal IV, which is the longest. Metacarpal V is 71% of the length of Metacarpal IV. The phalangeal formula is 2–3–5–5–3 for the manus, indicating presence of hyperphalangy in *Luopingosaurus* (see Discussion below).

In the pelvic girdle, the ilia, pubes and ischia are well exposed (Fig. 4c–f). The ilium is nearly triangular with a relatively long and tapering posterior process. The plate-like pubis is well constricted at its middle portion, with the medial portion nearly equal to the lateral portion. The obturator foramen is slit-like, located at the posterolateral corner of this bone (Fig. 4e). The ischium is also plate-like, having a relatively narrow lateral portion and an expanded medial portion that is notably longer than the medial portion of the pubis. The posterolateral ischial margin is highly concave. The posterior pubic margin and anterior ischial margin are moderately concave, and both together would enclose the thyroid fenestra. The femur is slightly longer than the humerus, with a constricted shaft and equally expanded ends (Fig. 4d). No internal trochanter is developed. The tibia is nearly equal to the fibula in length; the former is straight and thicker than the slightly curved latter. Two ossified tarsals, calcaneum and astragalus, are nearly rounded; the latter is significantly larger than the former. As in *Honghesaurus*^23^, the astragalus lacks a proximal concavity. The right metatarsals are well-preserved. Metatarsal I is the shortest and stoutest phalange with an expanded proximal end, and Metatarsal IV is the longest. Metatarsal II is nearly twice the length of Metatarsal I. Metatarsal III is slightly shorter than Metatarsal IV, and Metatarsal V is 76% of the length of Metatarsal IV. The phalangeal count is 2–3–4–6–4, which is complete judging from the appearance of the distal phalanges in the right pes (Fig. 4c).

## Discussion

**Figure 5:**
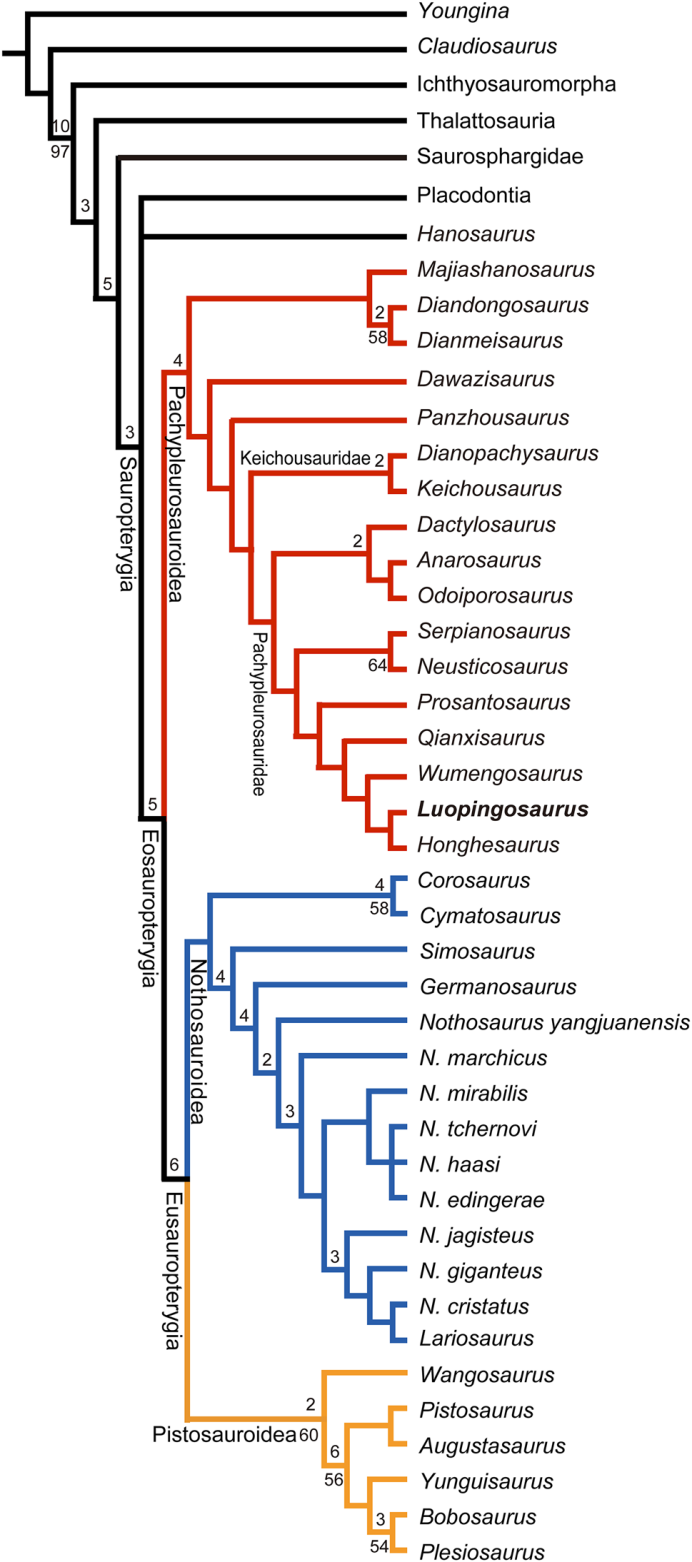
Phylogenetic position of *Luopingosaurus imparilis* gen. et sp. nov. Strict consensus of four trees rooted with *Youngina* (TL = 928, CI = 0.3211, and RI = 0.6836). Bremer decay indices larger than 1 and bootstrap values larger than 50% are indicated above and below the nodes of the tree, respectively.

Phylogenetic analysis recovered four most parsimonious trees (tree length = 928 steps, consistency index = 0.3211, retention index = 0.6836). In the strict consensus tree (Fig. 5), the ichthyosaurs and thalattosaurs are nested successively at basal positions relative to saurosphargids and sauropterygians (consistent with ref.^2^), and *Luopingosaurus* is recovered as a sister taxon of *Honghesaurus* within Pachypleurosauridae at the base of Eosauropterygia. Notably, the European pachypleurosaurids might be paraphyletic; *Prosantosaurus*^25^ forms a sister taxon to a clade comprising four Chinese taxa *Qianxisaurus*, *Wumengosaurus*, *Luopingosaurus* and *Honghesaurus*. The monophyly of this clade (Chinese pachypleurosaurid subgroup) is well supported by several synapomorphies (presence of a snout more than half of skull length, a retracted external naris, 27 or more dorsal vertebrae, and absence of a trough on dorsal surface of retroarticular process). Although *Prosantosaurus* was previously recovered as a sister taxon to a clade consisting of three other European taxa (*Serpianosaurus*, *Proneusticosaurus* and *Neusticosaurus*), the data matrix used in that analysis^25^ did not include any Chinese pachypleurosauroids. Our analysis would rather indicate a closer relationship of *Prosantosaurus* with Chinese pachypleurosaurids, as they share several derived features (exclusion of the premaxilla from the medial border of the naris, nasals not extending posterior to the level of the anterior orbital margin, a squamosal broadly separated from the ventral margin of the skull, and presence of a distinct expansion on the distal head of the sacral rib). *Luopingosaurus* shares with *Honghesaurus* two derived features (a retracted naris with a longitudinal diameter distinctly less than half the longitudinal diameter of orbit, and absence of a proximal concavity of the astragalus), but it is easily distinguished from the latter and other pachypleurosauroids with a series of autapomorphies (a long and anteriorly pointed snout, 55.0% of skull length; 21 cervical and 27 dorsal vertebrae; presence of hyperphalangy in the manus; and a short and stout Metatarsal I with an expanded proximal end).

The discovery of *Luopingosaurus* documents the oldest long-snouted pachypleurosaurid, providing new insights into the ecological adaption of this clade. The previously known long-snouted pachypleurosaurid *Wumengosaurus* is about one million years younger than *Luopingosaurus* and *Honghesaurus*^23^. In comparison, keichousaurids and some basal pachypleurosauroids (*Dianmeisaurus*, *Dawazisaurus* and *Panzhousaurus*) have a short snout with a ratio of preorbital length to skull length (PS ratio) ranging from 22.4% to 38.8% (Table 2); *Diandongosaurus* and most pachypleurosaurids (except *Luopingosaurus* and *Wumengosaurus*) have a moderately long snout (PS = 43.1–47.9%); *Luopingosaurus* has a noteworthily longer snout (PS = 55.0%) and *Wumengosaurus* the longest snout among this clade (PS = 63.8%). Analogous to crocodylians^43^, the divergence between short-snouted (brevirostrine) keichousaurids and relatively long-snouted (longirostrine) pachypleurosaurids was probably driven by ecological specializations related to feeding and foraging. Pachypleurosauroids generally have a flattened skull with the tooth row restricted to a level in front of the orbit, and the peg-like teeth seem to be designed for grasping prey with a quick sweeping bite^44^. The snout elongation apparently increases the length of the tooth row, which could be more effective for grasping prey and preventing prey from escaping. Additionally, the longer snouts in derived pachypleurosauroids have a more pointed anterior tip than those in basal forms, and they might have evolutionary advantage in chasing fast swimming preys because the pointed tip helps direct the flow of water over the body surface to reduce drag. Outside of pachypleurosauroids, a similarly anteriorly pointed snout associated with an elongate trunk is otherwise present in some thalattosaurs^45^, and this likely represents convergent adaptation driven by similar feeding and swimming mechanics.

**Table 2:** Measurements (in mm) for cranial bones of pachypleurosauroids with relatively complete skeletons.

Taxon	PL	SL	HL	ML	PL/SL	HL/ML	Specimen
*Dianmeisaurus gracilis*	6.1	24.0	4.6	27.0	25.4%	17.0%	IVPP V18630
*Diandongosaurus acutidentatus*	10.5	23.3	?	27.0	45.1%	?	IVPP V17761
*Diandongosaurus cf. acutidentatus*	29.0	63.3	10.9	69.0	45.8%	16.1%	WIGM SPC V1105
*Dawazisaurus brevis*	16.0	41.2	?	50.0	38.8%	?	NMNS000933-F034397
*Panzhousaurus rotundirostris*	4.5	20.1	?	23.0	22.4%	?	GMPKU- P-1059
*Keichousaurus hui*	6.8	23.3	3.8	26.4	29.2%	14.4%	NMNS CYN2005-12
*Dianopachysaurus dingi*	7.5	19.7	?	26.3	38.1%	?	LPV 31,365
*Anarosaurus pumilio*	18.5	38.0	?	47.0	48.7%	?	M4/12
*Neusticosaurus pusillus*	12.5	29.0	4.7	31.6	43.1%	14.9%	PIMUZ T3934
*Serpianosaurus mirigiolensis*	19.9	41.7	6.6	46.9	47.7%	14.1%	PIMUZ T3681
*Prosantosaurus scheffoldi*	21.9	48.0	?	58.5	45.6%	?	PIMUZ A/III 1274
*Qianxisaurus chajiangensis*	42.5	90.6	?	99.0	46.9%	?	NMNS-KIKO-F044630
*Honghesaurus longicaudalis*	15.8	33.0	?	38.9	47.9%	?	IVPP V30380
*Luopingosaurus imparilis*	23.5	42.7	4.5	46.2	55.0%	9.7%	IVPP V19049
*Wumengosaurus delicatomandibularis*	54.0	84.7	7.0	98.0	63.8%	7.1%	ZMNH M8758

HL, Hyoid length; PL, preorbital (snout) length; SL, skull length; ML, mandibular length; ?, unknown.

**Figure 6:**
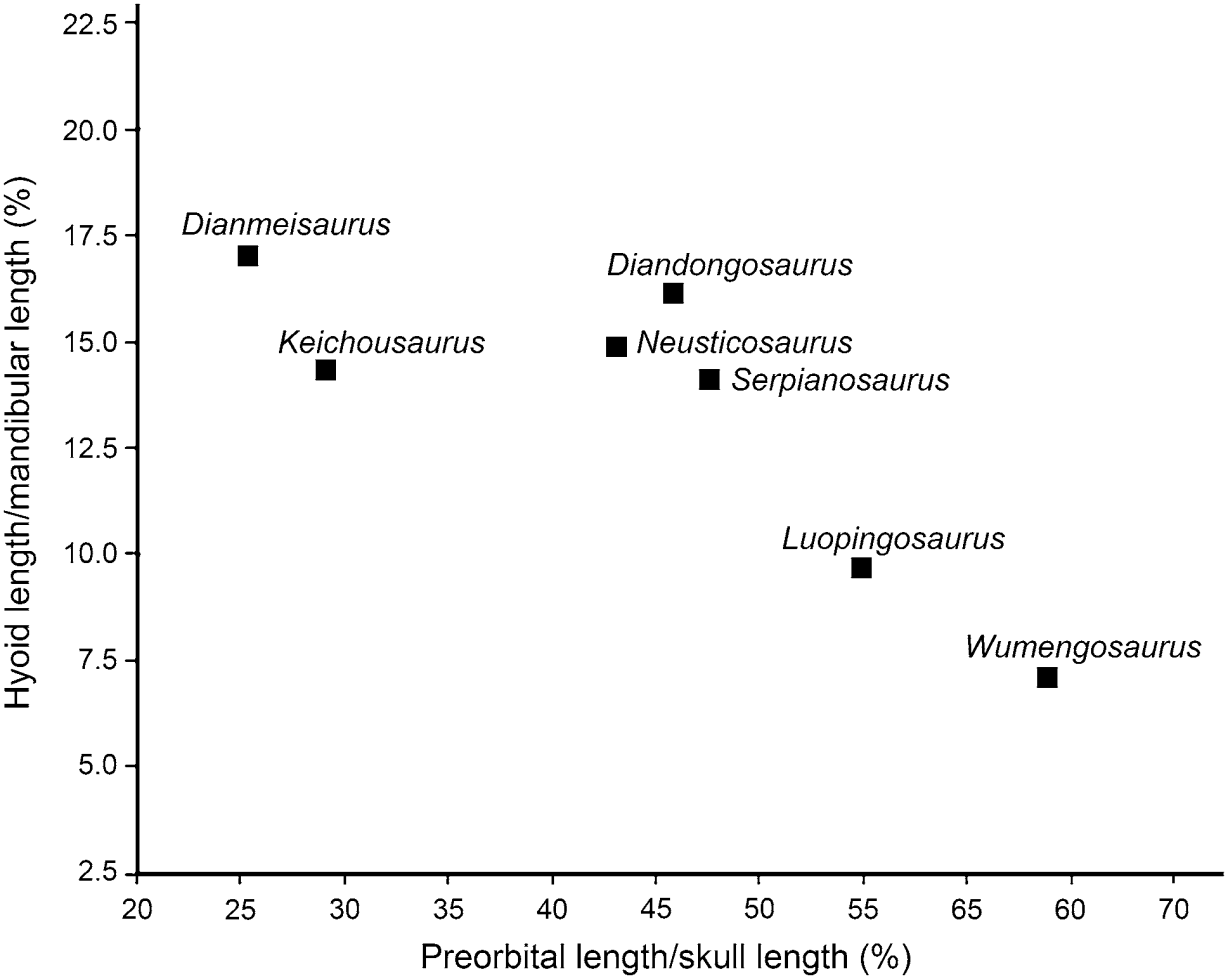
The ratios of preorbital length to skull length relative to those of hyoid length to mandibular length in Pachypleurosauroidea.

The discovery of *Luopingosaurus* provides new information on the hyobranchial apparatus and feeding adaption in pachypleurosauroids. The superb preservation of the holotype shows that *Luopingosaurus* has a pair of rod-like hyoids, which may have played an important role in securing prey because rapid depression and retraction of the hyobranchial apparatus following sudden opening of jaws would have created the suction necessary to draw the prey into the buccal cavity. Besides *Luopingosaurus*, well-developed hyoids are also known in some other pachypleurosauroids^8–12,18,22^, although they are unknown in the remaining members of this clade probably because of incomplete preservation. Based on this feature, Rieppel^44^ proposed that pachypleurosauroids likely employed suction as a primary solution to the biomechanical problems associated with feeding in a liquid medium, resembling cetaceans and many other aquatic vertebrates^46^. To trace the variation of hyoid bones in pachypleurosauroids, we conducted a survey of the ratio of hyoid length to mandibular length (HM ratio) across all pachypleurosauroids with hyoids preserved, and found that basal pachypleurosauroids have the highest HM ratios (17.0% in *Dianmeisaurus* and 16.1% in *Diandongosaurus*), keichousaurids and European pachypleurosaurids have moderate ratios of 14.1–14.9%, and Chinese pachypleurosaurids the lowest ratios of 7.1–9.7% (Table 2). Therefore, there is an evolutionary trend towards the reduction of the HM ratio in pachypleurosauroids. Notably, the reduction of the HM ratio is clearly associated with the increase of the snout length in this clade (Fig. 6). The snout elongation increases the efficiency for prey grasping but reduces the buccal cavity for suction. Studies of cetaceans^46^ have revealed that the species with the shortest, bluntest snout and smallest mouth gap generates greater negative pressures for suction feeding. This is likely also applied to pachypleurosauroids. The basal, short-snouted pachypleurosauroids with a higher HM ratio would have a higher suction efficiency than the derived, long-snouted forms with a lower HM ratio.

The discovery of *Luopingosaurus* also adds new information on the postcranial diversity of pachypleurosauroids. *Luopingosaurus* has 27 dorsal vertebrae among 48 presacrals. The most comparable counts are present in *Qianxisaurus* and *Wumengosaurus* (28 dorsal vertebrae) and *Honghesaurus* (29 dorsal vertebrae); by contrast, other pachypleurosauroids generally have 16–25 dorsal vertebrae. The ratio (43.8%) between the cervical and presacral vertebral numbers (CP ratio) in *Luopingosaurus* falls the range (CP = 0.39–0.47) of Pachypleurosauridae, contrasting higher CP ratios (0.50–0.58) in Keichousauridae and other pachypleurosauroids^23^. Although the tail is incompletely preserved, the long trunk and relatively slender humerus as in other derived pachypleurosaurids implicates that *Luopingosaurus* would have mainly relied on lateral undulation of the trunk and tail for aquatic propulsion^8–10^. Additionally, the count of caudal ribs of *Luopingosaurus* (six pairs) is slightly more than those of *Wumengosaurus* (three to five pairs) and the closely related *Honghesaurus* (five pairs) but fewer than those of other pachypleurosauroids (eight to 14 pairs). The reduction of caudal ribs in *Luopingosaurus*, similar to the condition in *Honghesaurus*^23^, make most of the tail laterally compressed. A functional advantage to this morphological adaption could be beneficial to maneuverability and energy efficiency for lateral undulatory swimming^47,48^.

Notably, *Luopingosaurus* is unique among pachypleurosauroids in having five phalanges in the third digit of the manus. The phalangal count (2–3-5–5–3) is slightly more than the plesiomorphic condition (2–3-4–5–3) for primitive amniotes^49^, representing an unexpected hyperphalangy (increase of phalanges) in this clade; no hyperphalangy has been recorded in other pachypleurosauroids^4,6–10,23,25^. A similar hyperphalangy (five phalanges in the third digit) is present in some other marine reptiles, e.g., the Early Triassic ichthyosaurian *Utatsusaurus hataii*^50^ and some Middle Triassic nothosaurids^51^, and extant North Atlantic right whale^52^. A slight difference is that the third digit in *Luopingosaurus* is shorter than the fourth one (which is the longest digit as in many other basal eosauropterygians), whereas in basal ichthyosaurians and North Atlantic right whale, the third digit is the longest one among five digits. Additionally, extreme hyperphalangy (exceeding the threshold value 4–6–6–6–6) have been evolved multiply times among extant and fossil aquatic amniotes (ichthyosaurs, plesiosaurs, mosasaurs and cetaceans) with a flipper limb morphology^49^. In most of these aquatic amniotes (except plesiosaurs), the flipper limbs are used as rudders for steering and stability^52^. So far, the hyperphalangy in *Luopingosaurus* represents the oldest record of this feature in sauropterygians. The hyperphalangy (limited to the third digit) in *Luopingosaurus* does not increase the flipper length, but may affect the contour of flipper deformation in flexion. As previously suggested for European pachypleurosaurids^8,9^ and *Honghesaurus*^23^, *Luopingosaurus* is likely an axial swimmer, and its propulsion is primarily provided by flexion of the body axis rather than by the flipper limbs. The functional advantage of the hyperphalangy is hard to know, but it may be advantageous to increase the flexion and extension of the digits, in response to hydrodynamic forces placed on the flipper in steering^52^.

The discovery of *Luopingosaurus* provides an importation addition for our understanding on the palaeobiogeographical evolution of pachypleurosauroids. The earliest and basal pachypleurosauroids are recorded from the Early Triassic of China (represented by *Majiashanosaurus*^17^), implicating an eastern Tethyan origin for the superfamily Pachypleurosauroidea. It was generally considered that the biogeographic evolution of pachypleurosauroids followed an east to west dispersal route^14,24,27^. In the early Middle Triassic, pachypleurosauroids diverged rapidly into two families, Keichousauridae and Pachypleurosauridae. As in basal pachypleurosauroids, keichousaurids were endemic to South China, but pachypleurosaurids emerged in Europe and diversified in both Europe and China. Although the family Pachypleurosauridae was previously considered endemic to Europe, the recent recoveries of pachypleurosaurids (*Honghesaurus* and *Luopingosaurus*) from Yunnan challenge this hypothesis and provide new insights into the Middle Triassic radiation of pachypleurosaurids. Phylogenetic studies incorporating *Luopingosaurus* described here resolve the similarly long-snouted *Wumengosaurus* as a derived pachypleurosaurid (rather than a basal pachypleurosauroid^24^ or a close relative of Ichthyosauromorpha^31,32^), and recover European pachypleurosaurids as successive sister groups to the Chinese pachypleurosaurid subclade (including *Wumengosaurus*, *Qianxisaurus*, *Honghesaurus* and *Luopingosaurus*), supporting a western Tethyan origin for this family^23^. The Pachypleurosauridae might disperse from west to east via the Palaeotethys Ocean in the Middle Triassic. As such, the biogeographic evolution of pachypleurosauroids could be bidirectional between the eastern and western Tethyan realms, more complicated than the previously thought^27^.

## Methods

The skull and pelvic girdle were scanned using the computed laminography scanner at the Institute of Vertebrate Paleontology and Paleoanthropology (IVPP), Chinese Academy of Sciences in Beijing, China, and the scan was set at the beam energy of 100 kV and flux of 50 μA with a resolution of 28.159 μm per pixel. To assess the phylogenetic position of *Luopingosaurus*, we incorporated it into a matrix expanded from a previous study^23^. The current data matrix includes 183 characters coded across 63 taxa (electronic supplementary material). The data matrix was generated by WinClada (v. 1.00.08)^53^. We used the basal diapsid *Youngina capensis* for out-group comparison. The maximum parsimony analyses were performed with a heuristic search in PAUP* (v. 4.0a169)^54^ using 800 random addition sequence replicates, holding five trees at each step, with the tree bisection and reconnection (TBR) strategy enabled and maxtrees set to automatically increase by 100. We measured the preorbital (snout), skull, hyoid and mandibular lengths for pachypleurosauroids, and imported these data into the paleontological statistics software package (PAST version 4.0^55^). The ratio of hyoid length to mandibular length (HM ratio, X axis) and that of preorbital length to skull length (PS ratio, Y axis) were plotted in a XY graph using the Landmarks of this software package.

## Supplementary Information

Supplementary Information.

## Data Availability

The data that support the findings of this study are available in the Supplementary Information. The nomenclatural acts for the new genus and species have been registered in the proposed online registration system (ZooBank) for the International Code of Zoological Nomenclature (http://zoobank.org/). The Life Sciences Identifier for this paper is urn:lsid:zoobank.org:pub: 21DE9931-FB6D-4225-B15C-6D359AE7E3CE.
