# Supplementary material for: A new long-snouted marine reptile from the Middle Triassic of China illuminates pachypleurosauroid evolution
Source: Sci Rep. 2023 Jan 5;13:16. doi: 10.1038/s41598-022-24930-y (PMC9816097; doi:10.1038/s41598-022-24930-y)
Supplement: Supplementary file 1 — Supplementary Information. [file 41598_2022_24930_MOESM1_ESM.pdf]

Supplementary Information for

A new long-snouted marine reptile from the Middle Triassic of China illuminates pachypleurosauroid evolution

Guang-Hui Xu[1,2], Qing-Hua Shang[1,2], Wei Wang[1,2], Yi Ren[1,2,3], Hong Lei[4], Jun-Ling Liao[5], Li-Jun Zhao[6], Chun Li[1,2]

[1]Key Laboratory of Vertebrate Evolution and Human Origins of Chinese Academy of Sciences, Institute of Vertebrate Paleontology and Paleoanthropology, Chinese Academy of Sciences, Beijing 100044, China
[2]CAS Center for Excellence in Life and Paleoenvironment, Beijing 100044, China
[3]University of Chinese Academy of Sciences, Beijing 100049, China
[4]Luoping Biota National Geopark, Land and Resources Bureau of Luoping County, Luoping 655800, China
[5]College of Economics and Management, Xingyi Normal University for Nationalities, Xingyi 562400, China
[6]Zhejiang Museum of Natural History, Hangzhou 310014, China

Table of Contents

1. Taxa and principal sources of data
2. Supplementary figures
3. Character list
4. Data matrix
5. References to supplementary information

1. Taxa and principal sources of data

GMPKU, Geological Museum of Peking University, Beijing, China

IVPP, Institute of Vertebrate Paleontology and Paleoanthropology, Chinese Academy of Sciences, Beijing, China

LPV, Luoping Biota National Geopark, Luoping, China

M, Institut für Geowissenschaften, Martin-Luther-Universität, Halle, Germany

NHMUK, Natural History Museum, London, UK;

NMNS, National Museum of Natural Science, Taichung, Taiwan, China

PIMUZ, Paläontologisches Institut und Museum, Universität Zürich, Zürich, Switzerland

WIGM, Wuhan Institute of Geology and Mineral Resources, Wuhan, China

ZMNH, Zhejiang Museum of Natural History, Hangzhou, China

Anarosaurus pumilio, Rieppel & Lin1995; M4/12
Dactylosaurus gracilis, Rieppel & Lin1995
Dawazisaurus brevis, Cheng et al., 2016; NMNS 000933-F034397
Diandongosaurus acutidentatus, Shang et al., 2011; IVPP V17761
Diandongosaurus cf. acutidentatus, Liu et al., 2021; WIGM SPC V1105
Dianmeisaurus gracilis, Shang & Li, 2015; IVPP V18630
Dianopachysaurus dingi, Liu et al., 2011; LPV 31365
Honghesaurus longicaudalis, Xu et al., 2022; IVPP V30380
Keichousaurus hui, Lin & Rieppel, 1998; Holmes et al., 2008; NMNS CYN2005-12
Luopingosaurus imparilis, IVPP V19049
Majiashanosaurus discocoracoidis, Jiang et al., 2014
Neusticosaurus pusillus, Sander, 1989; PIMUZ T3934
Odoiporosaurus teruzzii, Renesto et al., 2014
Panzhousaurus rotundirostris, Jiang et al., 2019; Lin et al., 2021; GMPKU- P-1059
Prosantosaurus scheffoldi, Nicole et al., 2022; PIMUZ A/III 1274
Qianxisaurus chajiangensis, Cheng et al., 2012; NMNS KIKO-F044630
Serpianosaurus mirigiolensis, Rieppel, 1989; PIMUZ T3681
Wumengosaurus delicatomandibularis, Wu et al., 2011; ZMNH M8758

2. Supplementary figures

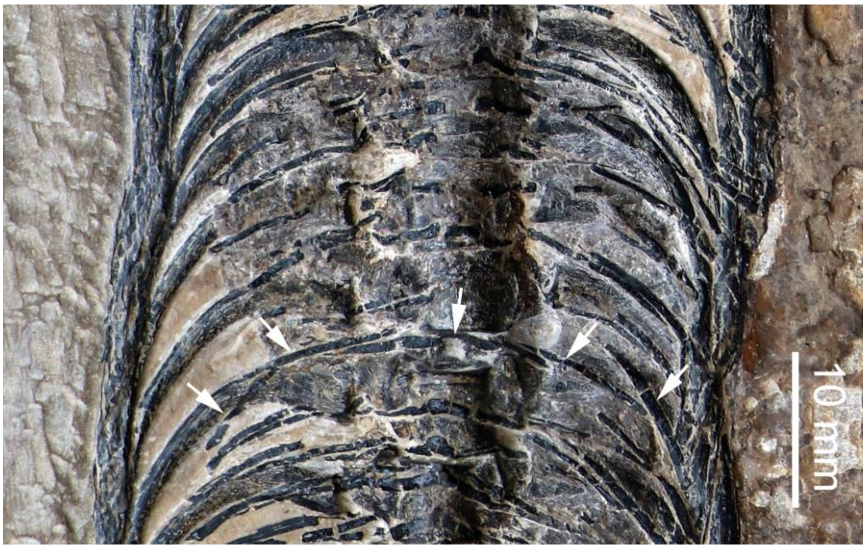

Figure 1S. The posterior trunk of *Luopingosaurus imparilis* gen. et sp. nov. (IVPP V19049), showing the pachyostosis in posterior dorsal ribs and five elements in each gastralium.

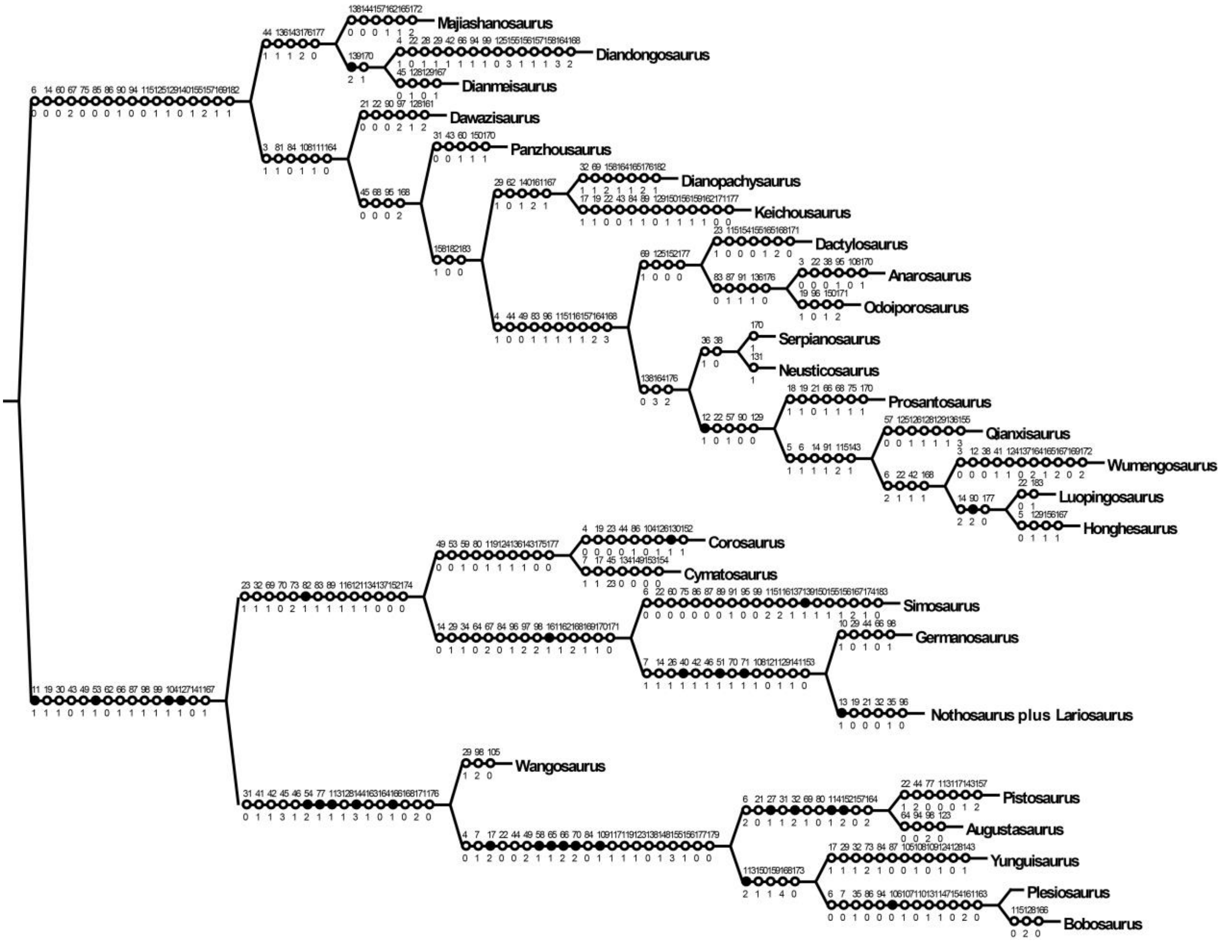

Figure 2S. Part of strict consensus of four most parsimonious trees, showing character optimisations within the Eosauropterygia. The topologies within *Nothosaurus* and *Lariosaurus* are omitted.

3. Character list

Of 183 characters, most (149) are taken from Lin et al. (2021) and Xu et al., (2022), which in turn based mainly on those of Rieppel and Lin (1995), Rieppel et al. (2002), Liu et al. (2011, 2014), Benson et al. (2012), Neenan et al. (2013, 2015), Li et al. (2014), Ma et al. (2015), Lin et al. (2019), and Li and Liu (2020). 34 newly added characters are marked with an asterisk; among them, Chars. 5 and 116 are two new characters proposed here, and others are adopted or slightly modified from Rieppel et al. (2002), Müller (2007), Li et al. (2011), Liu et al. (2013), and Ji et al. (2016). The original character sequences of Rieppel et al. (2002) are indicated at the end of each character description with an R before the number.

(1) Body contours (trunk): elongated (0); broad and rounded (1). (From Li et al., 2014:144)

(2) Dermal armour (‘osteoderms’): absent (0); present (1); forming carapace (2). (From Neenan et al., 2015: 138; Lin et al., 2021)

(3) Bones in dermatocranium: distinctly sculptured (0); relatively smooth (1). (From Rieppel and Lin, 1995:1)

(4) Preorbital and postorbital region of skull: of subequal length (0); preorbital region distinctly longer (1); postorbital region distinctly longer (2). (R12)

(5) Preorbital length in adults*: no more than half of skull length (0); more than half of skull length (1).

(6) Snout: relatively short (0); elongated with broad anterior termination (1); elongated and tapering anteriorly (2). (R132)

(7) Distinct snout constriction in adult: absent (0); present (1). (R3)

(8) Snout*: nearly horizontal (0); turned downwards (1). (Müller, 2007: 2; Liu et al., 2013: 1).

(9) Premaxillae*: small (0); large, forming most of snout in front of external nares (1). (R1)

(10) Premaxilla(e) in adult: paired (0); partly or fully fused (1). (From Liu et al., 2014:7)

(11) Ascending process of maxilla: distinct (0); reduced (low) (1). (From Li et al., 2014:138)

(12) Little contribution of premaxilla to the medial border of the external naris: absent (0); present (1). (From Xu et al., 2022: 149)

(13) Maxilla, depression at lateral margin of external naris and a foramen at its bottom for the exit of a lateral branch of superior alveolar nerve: absent (0); present (1). (From Liu et al., 2014:8)

(14) External nares: not retracted (0); retracted with a longitudinal diameter approaching or exceeding half the longitudinal diameter of orbit (1); retracted, narrow, and with a longitudinal diameter distinctly less than half the longitudinal diameter of orbit (2). (R133)

(15) Nasals in adult: paired (0); fused (1). (From Liu et al., 2014:9)

(16) Nasal(s): shorter than frontal(s) (0); longer than frontal(s) (1). (R5)

(17) Nasal(s): not reduced (0); reduced (1); absent (2). (R6)

(18) Nasal, anterolateral process lining the entire medial margin of external naris: absent (0); present (1). (From Liu et al., 2014:11)

(19) Nasal(s): meeting each other (0); separated from one another by nasal processes of the

premaxillae extending back to frontal bone(s) (1). (From Liu et al., 2014:10)

(20) Nasal anteriorly extending beyond external naris*: false (0); true (1). (Ji et al., 2016: 10)

(21) Nasal, length behind level of posterior margin of external naris more than twice of the maximal width: absent (0); present (1). (From Rieppel, 2001) The coding for *Hanosaurus* is changed from '?' to '1' (Wang et al., 2022). The coding for *Panzhousaurus* is changed from '0' to '1' based on personal examination on IVPP V30747.

(22) Nasal*: does not extend posterior to level of anterior margin of orbit (0); does extend posteriorly beyond this level (1). (Liu et al., 2013: 9)

(23) Nasal-prefrontal contact: present (0); absent (1). (From Liu et al., 2014:14)

(24) Lacrimal*: present (0); absent (1). (Modified from R9)

(25) Maxilla-prefrontal contact*: absent (0); present (1). (Ji et al., 2016: 5)

(26) Dorsal exposure of prefrontal: large (0); reduced (1). (R11)

(27) Prefrontal: without slender anteromedial process (0); with slender anteromedial process entering between maxilla and premaxilla (1). (R121)

(28) Frontal: participating in the formation of dorsal margin of orbit (0); excluded from dorsal margin of orbit by a contact of prefrontal and postfrontal (1). (R10)

(29) Frontal(s) in adult: paired (0); fused (1). (R14)

(30) Lateral edge of frontal: concave (0); rather straight or slightly convex (1). (From Rieppel and Lin, 1995)

(31) Distinct posterolateral processes of frontal(s): absent (0); present (1). (R15)

(32) Frontal: widely separated from upper temporal fossa (0); narrowly approaching upper temporal fossa (1); entering the anteromedial margin of upper temporal fossa (2). (R16)

(33) Postfrontal: with distinct lateral process overlapping the dorsal tip of postorbital (0); with reduced lateral process and hence more of an elongate shape (1). (Modified from R26; Lin et al., 2021).

(34) Postfrontal, distinct constriction behind the orbit: absent (0); present (1). (From Liu et al., 2014: 25)

(35) Postfrontal: entering upper temporal fossa (0); excluded from upper temporal fossa (1). (From Liu et al., 2014:26)

(36) Postorbital: in the formation of anterior margin of upper temporal fossa (0); excluded from anterior margin of upper temporal fossa by a contact of postfrontal and squamosal, or entering it narrowly (1). (From Lin et al., 2019:27)

(37) Postfrontal and postorbital*: separate (0); fused (1). (Liu et al., 2013: 15)

(38) Scleral ossicles*: present (0) or absent (1). (Rieppel and Lin, 1995: 21)

(39) Jugal: present (0); absent (1). (From Liu et al., 2014:21)

(40) Jugal: entering orbit (0); excluded from posterior margin of orbit (1). (From Liu et al., 2014:22)

(41) Jugal: excluded from upper temporal arch (0); entering upper temporal arch (1). (R25)

(42) Jugal-squamosal contact: absent (0); present (1). (From Liu et al., 2014)

(43) Distinctly open L-shaped (boomerang-shaped) jugal: absent (0); present (1). (From Neenan et al., 2015: 139)

(44) Parietal(s) in adult: paired (0); fused in their posterior part only (1); fully fused (2). (R17) The coding for *Hanosaurus* is changed from ‘2’ to ‘0’ (Wang et al., 2022).

(45) Parietal skull table: broad (0); weakly constricted (1); strongly constricted (at least posteriorly) (2); forming a sagittal crest (3). (R19)

(46) Parietal skull table, constriction in the posteriormost part: absent (0); present (1). (From Liu et al., 2014:19)

(47) Postparietals*: present (0); absent (1). (R20)

(48) Tabulars*: present (0); absent (1). (R21)

(49) Pineal foramen: close to the middle of skull table (0); weakly displaced posteriorly (1); strongly displaced posteriorly (2); displaced anteriorly (3). (R18; Lin et al., 2021)

(50) Pineal foramen located within a deep trough: absent (0); present (1). (From Liu et al., 2014)

(51) Temporal region of skull: relatively high (0); strongly depressed (1). (R4)

(52) Upper temporal fossa*: present (0); secondarily closed (1). (Modified from Li et al., 2011)

(53) Ratio of longitudinal diameters, upper temporal fossa to orbit: between 1.0 and 2.0 (0); 2.0 or more (1); less than 1.0 (2). (Modified from Liu et al., 2014)

(54) The anteromedial corner of upper temporal fossa: not (0); partially (1); fully floored by a descensus from postorbital, which together with neighbouring elements (postfrontal, parietal) separates it from orbit (2). (R122)

(55) Supratemporals*: present (0); absent (1). (R22)

(56) Lower temporal fossa: absent (0); present (1). (R27; Lin et al., 2021)

(57) Squamosal: descending to ventral margin of skull (0); broadly separated from ventral margin of skull (1). (R28)

(58) A box-like suspensorium of squamosal: absent (0); present (1). (R123)

(59) Distinct notch of squamosal to receive distal tip of paroccipital process: absent (0); present (1). (R32)

(60) Quadratojugal: present (0); absent (1). (R29)

(61) Anterior process of quadratojugal: present (0); absent (1). (R30)

(62) Posterior margin of quadrate: straight (0); concave (1). (R37)

(63) Quadrate*: covered by squamosal and quadratojugal in lateral view (0); exposed in lateral view (1). (R38)

(64) Dorsal wing of epipterygoid: approximately as broad as its base (0); narrower than its base (1). (R39)

(65) Braincase: located at posterior end (0); deeply recessed below parietal skull roof (or parietal sagittal crest) (1). (R124)

(66) Occipital crest: absent (0); present but squamosals not meeting behind parietal (1); present and squamosals meeting behind parietal (2). (R36)

(67) Occiput: with paroccipital process forming the lower margin of posttemporal fossa and

extending laterally (0); paroccipital processes trending posteriorly (1); plate-like with no distinct paroccipital process and with strongly reduced posttemporal fossae (2). (R31)

(68) Occiput (posterior margin of skull table): nearly straight (0); deeply concave (excavated) (1). (From Li et al., 2014:139)

(69) Mandibular articulations: approximately at level with occipital condyle (0); displaced to a level distinctly behind occipital condyle (1) (R33; Lin et al., 2021)

(70) Supraoccipital: exposed more or less vertically on occiput (0); exposed more or less horizontally at posterior end of parietal skull table (1); U-shaped (2). (R35)

(71) Supraoccipital: below the occipital exposure of the parietal (0); sutured with the parietal horizontally (1). (From Ma et al., 2015:141)

(72) Contact between exoccipitals and basioccipital condyle*: present (0); absent (1). (R34)

(73) Basioccipital tubera: free (0); in complex relation to pterygoid, as they extend ventrally (1); in complex relation to pterygoid, as they extend laterally (2). (R42)

(74) Vomer contact pterygoid: present (0); absent (1). (From Li et al., 2014:146)

(75) Premaxillae: entering internal naris (0); excluded from internal naris (1). (R45)

(76) Palate*: kinetic (0); akinetic (1). (R41)

(77) Posterior palatine vacuities (Andrews, 1896; distinct medial emargination [concavity] on quadrate ramus of pterygoid behind palatobasal articulation): absent (0); present (1). (R125)

(78) Suborbital fenestra*: absent (0); present (1). (R43)

(79) Pterygoids: longer than palatines (0); shorter than palatines (1). (R130)

(80) Pterygoid flanges: well developed and transversely oriented (0); well developed and longitudinally oriented (1); strongly reduced (2). (R44)

(81) Ectopterygoid: present (0); absent (1). (R46)

(82) Internal carotid passage: entering basicranium (0); entering quadrate ramus of pterygoid (1). (R47)

(83) Splenial bone: entering mandibular symphysis (0); excluded there from (1). (R52)

(84) Distinct coronoid process of lower jaw: absent (0); present (1). (R49)

(85) Strongly projecting lateral ridge of surangular defining the insertion area for superficial adductor muscle fibres on the lateral surface of lower jaw: absent (0); present (1). (R50)

(86) Mandibular symphysis: short (0); somewhat enforced (1); elongated and ‘scoop-like’ (2). (R51)

(87) Mandibular symphysis, anterior fusion: absent (0); present (1). (From Liu et al., 2014).

(88) Dorsal margin of dentary symphysis*: straight (0); recurved (1). (Liu et al., 2013: 21)

(89) Retroarticular process: short (0); long (1). (From Liu et al., 2014)

(90) Trough(s) on dorsal surface of retroarticular process: absent (0); single trough (1); two troughs (2). (Modified form Rieppel and Lin, 1995:23; Xu et al., 2022)

(91) Marginal teeth, lingual surface of crown: convex (0); concave (1). (From Li et al., 2014:149)

(92) Durophagous dentition: absent (0); present (1). (R128)

(93) Number of premaxillary teeth: four or more (0); three or less (1). (R129)

(94) Anterior (premaxillary and dentary) teeth: upright or only slightly procumbent (0); strongly procumbent (1); absent (2). (R54; Neenan et al., 2015)

(95) Premaxillary and anterior dentary fangs: absent (0); present (1). (R55)

(96) One or two enlarged teeth on maxilla: present (0); absent (1). (R56) Personal exmianton on IVPP V30746 indicates that one enlarged tooth is present on the maxilla of *Panzhousaurus*. Accordingly, the coding for this taxon is changed from '1' to '0'.

(97) Number of small maxillary teeth anterior to the maxillary fang(s): 3 or less (0); 4 (1); 5 or more (2). (Modified from Liu et al., 2014:46)

(98) Maxillary tooth row: restricted to a level in front of the posterior margin of orbit (0); extending backwards to a level below the posterior corner of orbit and/or the anterior corner of upper temporal fossa (1); extending backwards to a level below the anterior one third to one half of upper temporal fossa (2). (R57)

(99) Overbite*: (0) absent or very slightly; (1) present. (Ji et al., 2016: 50)

(100) Vomerine teeth*: present (0); absent (1). (Liu et al., 2013: 28)

(101) Palatine dentition: single row with four or more teeth (0); single row with three to one teeth/tooth (1); absent (2); multiple rows with small numerous teeth/denticles (3). (From Neenan et al., 2015:140)

(102) Teeth on pterygoid flange*: present (0); absent (1). (R58)

(103) Vertebrae*: notochordal (0); non-notochordal (1). (R59)

(104) Vertebrae: amphicoelous (0); platycoelous (1); procoelous/opisthocoelous (2). (R60)

(105) Cervical centra*: rounded ventrally (0); keeled ventrally (1). (R63)

(106) Cervical vertebrae, proportions of anterior cervical neural spines: taller than their anteroposterior length (0); longer than tall (1). (Modified from Benson et al., 2012)

(107) Rib facets of the anterior-middle cervical vertebrae: separated (0); co-jointed (1). (Modified from Benson et al., 2012)

(108) Vertebral centrum: distinctly constricted in ventral view (0); with parallel lateral edges (1). (R67)

(109) Subcentral foramina: absent (0); present (1). (R127)

(110) Zygosphene-zygantrum articulation: absent (0); present (1). (R64)

(111) Zygapophyseal pachyostosis: absent (0); present (1). (R69)

(112) Neural canal: evenly proportioned (0); distinctly higher than wide (1); wider than high (2). (From Li et al., 2014:140)

(113) Number of cervical vertebrae: 30 or below (0); more than 30 but less than 40 (1); 40 or more (2). (R134; Li et al. 2014)

(114) Parapophysis shifting backwards on centrum along cervical vertebral column: absent (0); present (1). (R135)

(115) Number of dorsal vertebrae: 20 or below (0); 21 to 26 (1); 27 or more (2). (Modified from

Rieppel and Lin, 1995:30; Li and Liu, 2020:106) The character state (2) has been revised from ‘28 or more’ to ‘27 or more’, for distinguishing *Luopingosaurus*, *Wumengosaurus*, *Honghesaurus* and *Qianxisaurus* from other pachypleurosauroids.

(116) CP ratio*: 0.38 or less (0); 0.39-0.49 (1); 0.50 or more (2).

(117) Distal articular surface on transverse processes of dorsal vertebrae: oblong (0); evenly rounded (1). (R136)

(118) Transverse processes of neural arches of the dorsal region: relatively short (0); distinctly elongated (1); absent (2). (Modified from R66; Motani et al., 2015) The character state (2) has been added for coding Ichthyosauriformes (Motani et al., 2015). The coding for *Hanosaurus* is changed from ‘?’ to ‘0’ (Wang et al., 2022).

(119) Distal end of transverse processes of dorsal vertebrae: not increasing in diameter (0); distinctly thickened (1). (R68)

(120) Sutural facets receiving pedicels of neural arch on dorsal surface of centrum in dorsal region: narrow (0); expanded into a cruciform or ‘butterfly-shaped’ platform (1). (R65)

(121) Anteroposterior trend of increasing inclination of pre- and postzygapophyses within dorsal and sacral region: absent (0); present (1). (R70)

(122) A distinct free anterior process of cervical ribs*: absent (0); present (1). (R71)

(123) Neural spines on dorsal vertebrae: low (0); tall (1). (From Liu et al., 2014: 48)

(124) Elongation of neural spines in proximal tail region: (0) absent; (1) present, spines as high as the corresponding vertebral length or higher. (Modified from Liu et al., 2014:49)

(125) Pachyostosis of dorsal ribs: absent (0); present (1). (R72) *Prosantosaurus scheffoldi* was described ‘no pachyostosis in ribs’, but the illustration of the holotype (Klein et al., 2002: Fig. 2F) shows distinct pachyostosis in posterior dorsal ribs. Therefore, it is coded as ‘1’ for this taxon.

(126) Distinct groove on the posterior aspect of the proximal shoulder region of the dorsal ribs: absent (0); present (1). (R120)

(127) Last dorsal rib: longer than first sacral rib (0); shorter than first sacral rib (1). (From Lin et al., 2019:98)

(128) Number of sacral ribs: three or less (0); four (1); five or more (2). (R73; Lin et al., 2017)

(129) Distinct expansion of distal head of sacral rib(s): present (0); absent (1). (R74)

(130) Sacral (and caudal) ribs or transverse processes and their respective centrum*: sutured (0); fused (1). (R75)

(131) Number of segments included in each gastral rib: five or more (0); three (1); paired (2). (Modified from Rieppel and Lin, 1995:34)

(132) Median gastral element: angulated (0); straight (1). (R131)

(133) Mineralized sternum*: absent (0); present (1). (R118)

(134) Medial gastral rib element: with a single lateral process (0); May with two-pronged lateral process (1). (R119)

(135) Lateral gastral rib element: straight laterally (0); bent upwards laterally (1). (From Li et al., 2014:143)

(136) Clavicles: broad medially (0); narrow medially (1). (R77)

(137) Clavicles: not meeting in front of interclavicle (0); meeting in an interdigitating anteromedial suture (1). (R79) The coding for *Hanosaurus* is changed from '?' to '0' (Wang et al., 2022).

(138) Anterolaterally expanded corners of clavicles: absent (0); present (1). (R80) The codings for *Qianxisaurus* and *Wumengosaurus* are changed from '1' to '0' based on Cheng et al. (2012) and Wu et al. (2011), respectively.

(139) Clavicle, anteriolateral process: absent (0); present, more medial (1); present, more lateral (2). (From Lin et al., 2019:109)

(140) Interclavicle: rhomboidal (0); T-shaped (1); triangular (2). (Modified from R82; Liu et al., 2014).

(141) Posterior process on (T-shaped) interclavicle: elongate or relatively short (0); rudimentary or absent (1). (R83)

(142) Scapula: represented by a broad blade of bone (0); with a constriction separating a ventral glenoidal portion from a posteriorly directed dorsal wing (1). (R84)

(143) Dorsal wing or process of eosauropterygian scapula: tapers to a blunt tip (0); ventrally expanded at its posterior end (1). (R85)

(144) Coracoid: of rounded contours (0); slightly waisted (1); strongly waisted (2); with expanded medial symphysis and ridge-like thickening of the bone extending from glenoid facet posteriorly along lateral edge of the bone (3). (R88) The coding for *Hanosaurus* is changed from '2' to '0' (Wang et al., 2022).

(145) Coracoid foramen*: enclosed by coracoid ossification (0); between coracoid and scapula (1). (R89)

(146) Pectoral fenestration*: absent (0); present (1). (R90)

(147) Limbs*: short and stout (0); long and slender (1). (R91)

(148) Foot: short and broad (0); long and slender (1). (R112);

(149) Humerus*: rather straight (0); 'curved' (1). (R92)

(150) Humerus: short than femur, or approximately of same length (0); longer than femur (1). (Modified from Lin et al., 2019:116) The coding for *Hanosaurus* is changed from '?' to '0' (Wang et al., 2022).

(151) Humerus, proximal width compared with the mid-shaft: greater (0); less (1). (From Liu et al., 2014:58)

(152) Deltopectoral crest: well developed (0); reduced (1); absent (2). (R93)

(153) Insertional crest for latissimus dorsi muscle: prominent (0); reduced (1). (R94)

(154) Epicondyles of humerus: prominent (0); reduced (1). (R95)

(155) Ectepicondylar groove on humerus: open notched anterior (0); open without notched anterior (1); closed (i.e. ectepicondylar foramen present) (2); absent (3). (R96) The coding for *Wumengosaurus* is changed from '0' to '1' (Wu et al., 2011).

(156) Entepicondylar foramen: present (0); absent (1). (R97) The coding for *Hanosaurus* is

changed from ‘?’ to ‘0’ (Wang et al., 2022).

(157) Radius, anterior (preaxial) margin of shaft: smoothly curved (0); concave (1); rather straight (2). (Modified from Lin et al., 2019:124)

(158) Radius: shorter than ulna (0); longer than ulna (1); approximately of same length (2). (R98);

(159) Ulna, mid-diaphysis: slender (0); broadened (1). (From Liu et al., 2014:64)

(160) Ulna, anterior margin: smoothly concave (0); with a tuberosity (1). (From Liu et al., 2014:66)

(161) Ulna, posterior (postaxial) margin: concave (0); rather straight (1); convex (2). (From Lin et al., 2019:128)

(162) Ulna, distinctly broadened proximal head compared with the radius: absent (0); present (1). (Modified from Rieppel, 2001)

(163) Distal end of ulna: not expanded, narrower than proximal part (0); distinctly expanded to at least the width of proximal part (1). (R126)

(164) Total number of carpal ossifications: five or more (0); four (1); three (2); one or two (3). (Modified from R137; Xu et al., 2022).

(165) Intermedium: rounded (0); rectangular elongate (1). (From Rieppel and Lin, 1995:44)

(166) Acetabular portion of ilium: broadened (0); rod-like (1). (From Li and Liu, 2020) The coding for *Honghesaurus* is changed from ‘?’ to ‘0’ (Xu et al., 2022: fig. 4).

(167) Spina praeacetabuli: absent (0); weakly developed (1); well developed (2). (From Li and Liu, 2020)

(168) Iliac blade: well developed (0); reduced but projecting beyond level of posterior margin of acetabular portion of ilium (1); reduced and no longer projecting beyond posterior margin of acetabular portion of ilium (2); absent, (i.e., reduced to simple dorsal stub) (3); elongated shaft (4). (Modified from R99; Neenan et al., 2015) The codings for *Hanosaurus* and *Honghesaurus* are changed from ‘?’ to ‘1’ (Wang et al., 2022; Xu et al., 2022), and the coding for *Wumengosaurus* is changed from ‘0’ to ‘1’ (Wu et al., 2011).

(169) Pubis: of rounded contours (0); waisted (1). (From Lin et al., 2019:133)

(170) Pubis: with convex ventral (medial) margin (0); with concave ventral (medial) margin (1). (R100; Li et al., 2014)

(171) Obturator foramen in adult: closed (0); open (1); absent (2). (R101; Li et al., 2014)

(172) Thyroid fenestra: absent (0); present (1); reduced (2). (R102; Li et al., 2014)

(173) Femoral shaft: stout and straight (0); slender and sigmoidally curved (1). (R104)

(174) Internal trochanter: well developed (0); reduced (1). (R105) The coding for *Hanosaurus* is changed from ‘0’ to ‘1’ (Wang et al., 2022).

(175) Intertrochanteric fossa: distinct but reduced (0); rudimentary or absent (1). (R106; Lin et al., 2021)

(176) Total number of tarsal ossifications: four or more (0); three (1); two or less (2). (R115)

(177) Proximal concavity of astragalus: absent (0); present (1). (R110) The coding for *Honghesaurus* is changed from ‘?’ to ‘0’ (Xu et al., 2022: fig. 4).

(178) Distal tarsal 1*: present (0); absent (1). (R113)

(179) Distal tarsal 5: present (0); absent (1). (R114)

(180) Metatarsal 5*: long and slender (0); distinctly shorter than other metatarsals and with a broad base (1). (R116)

(181) Metatarsal 5*: straight (0); 'hooked' (1). (R117)

(182) Pes, ungual phalanges extremely expanded: absent (0); present (1). (From Lin et al., 2019:145)

(183) Hyperphalangy in manus: absent (0); present (1). (From Rieppel et al., 2003) The coding for *Hanosaurus* is changed from '?' to '0' (Wang et al., 2022).

4. Data matrix

Youngina

0011100000000200000010000000001000000000000000000000020010000011?000000010000010
000?100000000001001?00030001100000000000000000000000??01001000100?00?110000000011
0000000000000001010000000

Claudiosaurus

0001000000000000000000100000000010000000000001000110000200100001 00?0000000?0000010
20010?0000000001?000301011000000000000000?0000000002000010001 00?010110001111020
0000000000000001010000000

Nanchangosaurus

010112001000 0?0100000100??0000110010010 00??0001130002?0110?01?0?0000010??????0?
?????????0??0?????????????1???00?00100000000 11000?00??0????????0?0100?0?011131?2
0000100???????????????000

Hupehsuchus

01011200100002010000010011010 0?100000?0001?0??11?000200110?0????0??00?0??0???0?
????0?1000??0?201?????????1???00?0010000000 11000?00100010000??0?0000001011 13122
0000100?04000?0120?010000

Cartorhynchus

00000010100002010001010000000000000000000011010113 0002?0110?0110?00001?0??????0?
???0000000??1?201??0????0?1?1000???1002000?00100?00??0?00000100????000101113112
0?00110??400?0012?????000

Chaohusaurus_geishanensis

000112101000020100011100110100110010 00000?10101100002001100011 1?000011010???00?
2?00001000?010001?00?2?1011?1000?0?2002000010000???100000?00100?110000101113112
0000000004001001220110000

Anshunsaurus

00111200100002001020010111000 11?01??110000000001130012001 0001?11?0011110?00?0010
0000111000000001?00121?01100000?00200000011110000 02?0?00000100?000000001113112
0000000000100001101010000

Xinpusaurus

0011120110000200112000111100011?01??10000000001100012001 1001?11?0011100?0??0010
?0??1100100010001?000201011?0000?002000000?11000?00??0??0000??0?000000001113112
00000??????0000110?010000

Concavispina

0011?20?10000?00112?0001110000??01??10000000011?00120011001?11?00111?0?0??00?0
?0??110??1000000??00?20?011?0000?002000000?11?0010010000000100?000000001113112
00000????0102001121110000

Sinosaurosphargis

110001001000020000000101110000 1001??11001112001130012?110001??0?0?11110??0?10?0
20?10?1000?100001?001?1?001?0000200?0011???0????????0?0010101?1?01?0?1?0?11??21
0000000???????????????0?0

Largocephalosaurus

0100110010000200000001011100001101100100111000113 0002?010001??0?0011110?????0?0
20?1011000010000 1?001?11001?00?000010011???00?0?000?00001010111?011001101110121
0000000001001001201010000

Paraplacodus

000001?0100002??01?1000110?1?1??00000100001??011??00001110?1100?0???0????1?1001
????112?001011101?0010110???00?010?101100?10100?00001?111000??1?11?00110010?112
0000110003101210011???00?

Placodus

0102011010000111010101011001(01)11000000100110(02)0011000000101001110100110 0011
1110011000102001001110 1?00111001110000100001100110100000001001100010 0?011001101
1001020000030021000210020110000

Cyamodus

12020110100001(01)000000001100001(01)000100100100200113000001010000100001 1100?0
111001110?1020000011001?00111?0?1?000010000?1000?000??000?1?0(01)?000?10?0??0??
?0110??12000002?01100120????1?00??

Psephoderma

1202021010000200111000011100010000100100100210113000001010100100000111 00??101001
11?????000?01?201?00111?011?0??0?0??0?10?010000?000?1001??????0?1?1001100102120
00000??01100121001111000?

Hanosaurus

00020000???00?010?0011011000101 0?000?10000?00?1130002011000???0?00?1110????1?0?
????????00?00??01?00??1?00??10??????0?0????0?1????0000101000????0110010???1?0??
?????????1001011011 1100?0

Majiashanosaurus

00???
???????????????????????10???00?????0?000?????1?0010?000011000111011001001?11002

0001021???10121?????100??

Anarosaurus

0001000010000000000001001100000100000000000010001100002011000??1110020110??001000
21?000010011000011?001211011000110001100010100000(01)10000001110(01)11(01)211001
00011101100000200031111110011000?

Dactylosaurus

0011000010000000000011111000001000000100001000110000201100??1?10020110??001000
21?1000000100001?00121101101011000010001010000?0100000000110??10211001?00100011
0000021002100l?1?1011?000

Prosantosaurus

00110000100100000110000110000010000000?00001000110000201110001?1??121010??011000
21??000000?000001?0012110110l011?00110001010010000000000001000110211001(01)0?111
0110000030003111111121110000

Serpianosaurus

00110000100000000000110110000010000100000010001100002011000011110020010??001000
21?1000000100001?00121101101011000110001010 0(01)00(01)1000000010001102110 01(01
)0(01)11101(12)000003000311111121110000

Neusticosaurus

00(01)100001000000000(01)0110110000(01)1000010000001000110000201100001111002001
0??00100021?(01)0000001000001?00121101101011000110001010100(01)10100000100(01)
110211001(01)0(01)1(01)(01)01100000(23)(01)00310(01)1111(12)1110000

Odoiporosaurus

001100001000000000101101100000100000010000100011000020110000111?00?01??????????
????00??00?100000?00???10???10110????0001010000???000000??10????211001100111011
00000??0031021111????????

Luopingosaurus

00?1120010?1?2000?0??00???0000??00??01000110??11???02?11?000111????00????0?1000
21??000000?100001?00121?011010110002l000??1??10000000000001000111211001001 11?011
00000300011011111120110001

Honghesaurus

0011020010010200000011011000001000000100011000110000201110 0??11?0020010????????
????00??002?00001?00???1??10?0?1?0021000?0100100010???????00??11???001001111111
000003001l????11120110000

Wumengosaurus

0001120010000100000011011000001000000000111000110000201110 00111?00?00?????0???0
21??00??000100001?0012110?101011?0021000101011000000 0?000000011121?001(01)01111
0110000021021001211121110 0?0

Panzhousaurus

00120000100000000000110110000000000000100000200113000201100011?1?0020010?????0?0

2???00??001000000?00?2110?10??110000200010100 1(01)001000000???0??10211001101111
0220000000000211111111(01)1110011

Dianopachysaurus

0012000010000000000011011000101100000100001200113000201100?0?01?00?01?0????????
????00??001000000000??????1????1?0?02?00?010010001 0?0?000?10??10???00100?111022
00200110121?1?1112111001?

Dawazisaurus

00120000100000000000000110000010000001000012101130002011000???1?00?1010????????
????00??000000010200??????1???11?0002?00?010010011 0???????10??10???00100?111022
0020000001????1111111001?

Diandongosaurus

000100001000000000000?0011001101000000100011110113000201100001111012101 0??0010?0
20?0100000100011000112 11?1100010?0002000101000?00100000011120111211001001113111
00000300021111111120110011

Dianmeisaurus

000200001000000000000110110000010000001 00001(12)00113000201100 0??11?0021010??001
00020?01000001000010000 1211?1100010?0002?00?0100100100000001112011121100100 1111
0220000020011111111120110011

Keichousaurus

0012000010000000101010011000101000000100000200113000201100001 01?00200101?001000
21?01000011000(01)00?001211011010110000200010100100000000000110111021100110(01)
(01)111211021000012100111110110000

Qianxisaurus

001111001001010000000100110000010000001000010001100002011000011 1?0020010????????
????00???00100001?00???10?1010?1?0021000?010001011000?001?00??112??00100?113011
00000300031?111112111000?

Simosaurus

0002000010100000001010(01)1100011(01)(01)010001000002101110001011000010100121 10
0120010002011010000010010 1?20121111??0010000220001110000?000000100111101021 1001
100111112001102002211011111111 0000

Germanosaurus

0002011011100100001011111100011101000101?1011111010101100???01?00?1???????10??
????????0??000111?1112???
?????????????????????????

Nothosaurus_yangjuanensis

000201101010110000000101110011100110010101021111101010110001101?0121111??011000
20???1??010000110221121 1111?101000011000101000(01)1110000100010011?211001000010
0121101010012110110111110001

Nothosaurus_marchicus

0002011010101100010001111100111001(01)00101010221111010101100011 01?0120011?2011
000201?01210100001102211211111?10110000100010 10?00?01000010???0??1?211001010010
0120011020012110110011 1100??
Nothosaurus_mirabilis
000201101010110001001111110011101010010(01)01022(01)11101010110000101001210 11?2
011000201101210100001101211211 1???10110?0?20001011000??10000?0011011102110011 00
0100??001?0??0121111100??1?00??
Nothosaurus_giganteus
000201101010110001(01)01111110011001 0?0010100021(01)1110101011000110 1?0121111??
0110000011012101000011012112111 11?101100011000101 0000?2100001010100110211001001
0110121011010012111110011110000
Nothosaurus_tchernovi
000201101010010001001101110011?010?0011????221112?101011000??01?0120011?20?1000
201??12?0??00011012112111???10110????0001011???????????????????????????000110??
??????????????1??????????
Nothosaurus_haasi
0001011010100000001011111100111000000 11????221112010101100011010012 0011?2011000
20??????0??00011002112 1???????110????000???1???????????????????????????100110??
??????????????100????????
Nothosaurus_jagisteus
000201101010110001 00??111100110000100101000221111010101100 0?101?0120011?20?1000
00?1012101000011012112111110101 1000?10001010000????00?10001010102 11001?00011010
0001020???111????????????
Nothosaurus_edingerae
0002011010100100011001?111001110100001?????2211111101011000??01?0120011?2011000
20??????0??000110?21121??
?????????????????????????
Nothosaurus_cristatus
00020110101011000100111111001100001101010002311120101011000 1?0100120011?2011000
00??????0??0001100211?1??
?????????????????????????
Lariosaurus_winkelhorsti
0002011011101210000011111100110000100101010200110010001100011010012001??2??1?0?
00??????0??000110011121??
?????????????????????????
Lariosaurus_juvenilis
000201101010120001001101110011100000010100021011101000110 00?1010012011 1?2011000
????????0??0001?0021121??
?????????????????????????

Lariosaurus_youngi

000201101010120000001101110011000010010100022011111O(01)0110001101?0120011120?1
00000?(01)012(01)010000110(01)2112111110101100011000101001011100000(01)000(01)0(
02)11?2110010(01)111001211010000031001101111110001

Lariosaurus_hongguoensis

000201101010120001OO??1111001110001OO11?0002101111100011000110l?012001??20?1000
?0?101200100001100211211111?10???00?1???????0??????00?00??0???1?2110010111110O2
1011000???10111112011OOO1

Lariosaurus_vosseveldensis

000201101110121001000101110111100010010100021111101000110001101?0120011????1?0?
?????1??0?0000010??11?1???
????????????????????????

Lariosaurus_buzzii

00020110111012100000111111001110000001?????2111100100011000??01?0120011?2011000
20?10120010000110(01)211211111?1011000?10001010000??10000001?00??11211001000111
11110110?00031011111??1?????

Lariosaurus_curionii

0002010010101200011O???11100111010100 11?0?0211111010001100 0??01?0120011?20?1000
20?1012?010000110021121111101011000??0001?10010?????????01102110211????11111010
11010??????????????1?????

Lariosaurus_balsami

00020110101010000000???11100111010?001000?022111(12)?100011000??01?0120011?2011
00020?10120010000110?2112111110101100011000101001012100 0?00011001102110010111 11
0121011000??310111110111 0001

Lariosaurus_calcagnii

0002011010101200000 00??011100111010000 10(01)0002211120100011000??0100120011?2011
00020?1012001000011 0?2112111110101100011000101010012100 0?001010211021100111111 0
012111100000311111111110001

Lariosaurus_valceresii

000201101010120000000101110011001000011?00022111(12)0100011000??01?0120011?2??1
?0?????01??0100001100211?11111?1011?001100010100001210000 00??10??10211001011110
01211010000031??111121110001

Lariosaurus_xingyiensis

000201101010120001001101110011000010011?00022111201000110001101?0120011?20?1000
00?10120010000110021121111101011?00110001010110121000000000002110211001011110012
11110000031??1100??1?00?1

Corosaurus

000001001010?2000000110110000111000001??0?0010110000001100 1??011011110?1???1000
00??111?0100001100 1??2110110001000011001111010110010001010101011211001001110012

00000200110011100101 1?00?

Cymatosaurus

000201101?1002001010111 1100(01)011(12)00000100000(12)(23)01100000001 10011?01?0(0
1)11100??0110000011??210?0000110011 12111?10001?0????0011??0?00???0?0000????????
?????0?000000?????00??01??011100????????

Wangosaurus

00020100101002000010110110001100000001001102311110001211000110??01110101???1?0?
????11??00000?1100211?11?010?01001??20001?100001100??0????10???0?1?00100?11001?
0??0?00???????1??01?10001

Pistosaurus

00000210101002?02?10?1?1101001120000010011023111 2?00121101?1?01112111????0?1000
00??????0??00011001112111?100110001?20011?11000???0?00?0??????1131?0?100?113122
00001??1(12)0????11?????00??

Augustasaurus

00000210101002??2?1000?110100112000001001?0031112000121101?1?010121112011011100
00??0121000000010021121111 1001?0011121011?1000????000?00?00??103?1??1?02113102
0000120??????????????????

Yunguisaurus

000001101010020010101001100011010000010??10031??200012?101?1?01112110?0?20??100
200011200000001100111211(01)010101002?1210?1?11100?00000?000??0??1131?011101113
112100010011400210 1100?00001

Plesiosaurus

0000000010100 2??2?10???1100001000010010011003111200012110101?0111211020?1011100
20??01010000000100111211(01)101010002012101 1011000?100100?00100??1031?111101103
11210200001140021011 00?00001

Bobosaurus

00??
???????????????????????1(01)10101000?00??0?1?110001200?0000?????????????1?0(12)
?0?1?????????01?0021???????????

5. References

Benson, R., M. Evans, and P. S. Druckenmiller. 2012. High diversity, low disparity and small body size in plesiosaurs (Reptilia, Sauropterygia) from the Triassic–Jurassic boundary. PLOS ONE 7:e31838.

Cheng, Y.-N., X.-C. Wu, T. Sato, and H.-Y. Shan. 2012. A new eosauropterygian (Diapsida, Sauropterygia) from the Triassic of China. Journal of Vertebrate Paleontology 32: 1335–1349.

Holmes, R., Y. N. Cheng, and X. C. Wu. 2008. New information on the skull of *Keichousaurus hui* (Reptilia: Sauropterygia) with comments on sauropterygian interrelationships. Journal of

Vertebrate Paleontology 28:76–84.
Hu, S.-X. et al. 2011. The Luoping biota: exceptional preservation, and new evidence on the Triassic recovery from end-Permian mass extinction. Proceedings of Royal Society B **278,** 2274–2282.
Ji, C., D.-Y. Jiang, R. Motani, W.-C. Hao, and Z.-Y. Sun. 2016. Phylogeny of the Ichthyopterygia incorporating recent discoveries from South China. Journal of Vertebrate Paleontology 36: e1025956.
Jiang, D.-Y., M. W. Maisch, W.-C. Hao, Y.-L. Sun, and Z.-Y. Sun. 2006. *Nothosaurus yangjuanensis* n. sp. (Reptilia, Sauropterygia, Nothosauridae) from the middle Anisian (Middle Triassic) of Guizhou, southwestern China. Neues Jahrbuch für Geologie und Paläontologie, Monatshefte 2006:257–276.
Jiang, D.-Y., Lin, W.-B., Rieppel, O., Motani, R. and Sun, Z.-Y. 2019. A new Anisian (Middle Triassic) eosauropterygian (Reptilia, Sauropterygia) from Panzhou, Guizhou Province, China. Journal of Vertebrate Paleontology 38:e1480113.
Li, C., D.-Y. Jiang, L. Cheng, X.-C. Wu, and O. Rieppel. 2014. A new species of *Largocephalosaurus* (Diapsida: Saurosphargidae), with implications for the morphological diversity and phylogeny of the group. Geological Magazine 151:100–120.
Li, C., O. Rieppel, X.-C. Wu, L.-J. Zhao, and L.-T. Wang. 2011. A new Triassic marine reptile from Southwestern China. Journal of Vertebrate Paleontology 31, 303–312.
Li, Q., and J. Liu. 2020. An Early Triassic sauropterygian and associated fauna from South China provide insights into Triassic ecosystem health. Communications Biology 3:63. doi: 10.1038/s42003-020-0778-7.
Lin, K. and O. Rieppel. 1998. Functional morphology and ontogeny of *Keichousaurus hui* (Reptilia, Sauropterygia). Fieldiana Geology 39:1–44.
Lin, W.-B., D.-Y. Jiang, O. Rieppel, R. Motani, C. Ji, A. Tintori, Z.-Y. Sun, and M. Zhou. 2017. A new specimen of *Lariosaurus xingyiensis* (Reptilia, Sauropterygia) from the Ladinian (Middle Triassic) Zhuganpo Member, Falang Formation, Guizhou, China. Journal of Vertebrate Paleontology. doi: 10.1080/02724634.2017.1278703.
Lin, W.-B., D.-Y. Jiang, O. Rieppel, R. Motani, C. Ji, A. Tintori, Z.-Y. Sun, and M. Zhou. 2021. *Panzhousaurus rotundirostris* Jiang et al., 2019 (Diapsida: Sauropterygia) and the recovery of the monophyly of Pachypleurosauridae. Journal of Vertebrate Paleontology. doi: 10.1080/02724634.2021.1901730.
Lin, W.-B., M. Zhou, and D.-Y. Jiang. 2019. Systematic study of the eosauropterygians from the Triassic of South China. Science Press, Beijing, China, 154pp. [Chinese]
Liu, J., L.-J. Zhao, C. Li, and T. He. 2013. Osteology of *Concavispina biseridens* (Reptilia, Thalattosauria) from the Xiaowa Formation (Carnian), Guanling, Guizhou, China. Journal of Paleontology 87:341–350.
Liu, J., O. Rieppel, D.-Y. Jiang, J. C. Aitchison, R. Motani, Q.-Y. Zhang, C.-Y. Zhou, and Y.-Y. Sun. 2011. A new pachypleurosaur (Reptilia: Sauropterygia) from the lower Middle Triassic

of southwestern China and the phylogenetic relationships of Chinese pachypleurosaurs. Journal of Vertebrate Paleontology 31:291-302.

Liu, J., S.-X. Hu, O. Rieppel, D.-Y. Jiang, M. Benton, N. Kelley, J. Aitchison, C.-Y. Zhou, W. Wen, J.-Y. Huang, T. Xie, and T. Lv. 2014. A gigantic nothosaur (Reptilia: Sauropterygia) from the Middle Triassic of SW China and its implication for the Triassic biotic recovery. Scientific Reports 4:7142. doi: 10.1038/srep07142.

Liu, Q.-L. *et al.* 2021. An injured pachypleurosaur (Diapsida: Sauropterygia) from the Middle Triassic Luoping Biota indicating predation pressure in the Mesozoic. Scientific Reports 11:21818.

Ma, L.-T., D.-Y. Jiang, O. Rieppel, R. Motani, and A. Tintori. 2015. A new pistosauroid (Reptilia, Sauropterygia) from the late Ladinian Xingyi marine reptile level, southwestern China. Journal of Vertebrate Paleontology. doi: 10.1080/02724634.2014.881832.

Motani, R., D.-Y. Jiang, G.-B. Chen, A. Tintori, O. Rieppel, C. Ji, and J.-D. Huang. 2015. A basal ichthyosauriform with a short snout from the Lower Triassic of China. Nature 517:485–488.

Müller, J. 2007. First record of a thalattosaur from the Upper Triassic of Austria. Journal of Vertebrate Paleontology 27:236–240.

Neenan, J. M., C. Li, O. Rieppel, and T. M. Scheyer. 2015. The cranial anatomy of Chinese placodonts and the phylogeny of Placodontia (Diapsida: Sauropterygia). Zoological Journal of the Linnean Society 175:415–428.

Neenan, J. M., N. Klein, and T. M. Scheyer. 2013. European origin of placodont marine reptiles and the evolution of crushing dentition in Placodontia. Nature Communications 4:1621.

Nicole, K., F. Heinz, E. Iris, T. L. Marta, R. Henning, and M.S. Torsten. 2022. A new pachypleurosaur from the Early Ladinian Prosanto Formation in the Eastern Alps of Switzerland. Swiss Journal of Palaeontology 141:12.

Renesto, S., G. Binelli, and H. Hagdorn. 2014. A new pachypleurosaur from the Middle Triassic Besano Formation of Northern Italy. Neues Jahrbuch für Geologie und Paläontologie - Abhandlungen 271:151–168.

Rieppel O. 2001. A new species of *Nothosaurus* (Reptilia: Sauropterygia) from the upper Muschelkalk (lower Ladinian) of southwestern Germany. Palaeontographica Abteilung A 263:137–161.

Rieppel, O., and K.-B. Lin. 1995. Pachypleurosaurs (Reptilia: Sauropterygia) from the Lower Muschelkalk, and a review of the Pachypleurosauroidea. Fieldiana Geology 32:1–44.

Rieppel, O. 1989. A new pachypleurosaur (Reptilia: Sauropterygia) from the Middle Triassic of Monte San Giorgio, Switzerland. Philosophical Transactions of the Royal Society of London. Series B 323:1–73.

Rieppel, O., P. M. Sander, and G. W. Storrs. 2002. The skull of the pistosaur *Augustasaurus* from the Middle Triassic of northwestern Nevada. Journal of Vertebrate Paleontology 22:577–592.

Sander, P. M. 1989. The pachypleurosaurids (Reptilia: Nothosauria) from the Middle Triassic of Monte San Giorgio (Switzerland) with the description of a new species. Philosophical

Transactions of the Royal Society of London. Series B 325:561–666.

Shang, Q.-H. and Li, C. 2015. A new small-sized eosauropterygian (Diapsida: Sauropterygia) from the Middle Triassic of Luoping, Yunnan, southwestern China. Vertebrata Palasiatica 53:265–280.

Shang, Q.-H., X.-C. Wu, and C. Li. 2011. A new eosauropterygian from Middle Triassic of eastern Yunnan Province, southwestern China. Vertebrata Palasiatica 49:155–171.

Wei, W., Q.-H. Shang, L. Cheng, X.-C. Wu, and C. Li. 2022. Ancestral body plan and adaptive radiation of sauropterygian marine reptiles. BioRxiv preprint doi: https://doi.org/10.1101/2022.04.25.489368.

Wu, X.-C., Y.-N. Cheng, C. Li, L.-J. Zhao, and T. Sato. 2011. New information on *Wumengosaurus delicatomandibularis* Jiang et al. 2008 (Diapsida: Sauropterygia), with a revision of the osteology and phylogeny of the taxon. Journal of Vertebrate Paleontology 31:70–83.

Xu, G.-H., Y. Ren, L.-J. Zhao, J.-L. Liao, and D-H. Feng. 2022. A long-tailed marine reptile from China provides new insights into the Middle Triassic pachypleurosaur radiation. Scientific Reports 12:7396.
